# Supplementary material for: Response process validity of three patient reported outcome measures for people requiring kidney care: a think-aloud study using the EQ-5D-5L, ICECAP-A and ICECAP-O
Source: BMJ Open. 2020 May 15;10(5):e034569. doi: 10.1136/bmjopen-2019-034569 (PMC7232621; doi:10.1136/bmjopen-2019-034569)
Supplement: Supplementary data [file bmjopen-2019-034569supp002.pdf]

Kidney patients' views on quality of life questionnaires
Participant Information Sheet

Information about the Research

We would like to invite you to take part in a research study that aims to understand how quality of life is measured in patient reported questionnaires. The study will be carried out by researchers from the University of Bristol together with the UK Renal Registry. To help you decide if you wish to take part, this leaflet explains the purpose of the research and how you would be involved. A member of our team will go through this information sheet with you and answer any questions you may have. This is likely to take about five to ten minutes.

What is the purpose of the study?

The aim of this study is to find out about what patients receiving kidney care think when answering quality of life questionnaires. Three quality of life measures are potentially useful for comparing the cost-effectiveness of different kidney care services. We are interested to know which measure you think best measures your quality of life.

Why have I been invited?

You have been invited because you are attending a clinic at the Richard Bright Renal Unit, Southmead Hospital Bristol. We hope that about 25 patients will take part.

Participation in the study is entirely up to you, but your help would be much appreciated. We will discuss the study with you and go through this information sheet before you make a decision. If you agree to take part, we will ask you to sign a consent form. One of our researchers will then arrange to interview you. You are free to withdraw from the study at any time and you do not need to tell us why. Please be assured that if you withdraw it will in no way affect the standard of care you receive.

Mitchell PM, *et al. BMJ Open* 2020; 10:e034569. doi: 10.1136/bmjopen-2019-034569

What will I have to do?
First we will ask you a few questions about you and the type of kidney care you receive. We will then ask you to 'think aloud' as you complete three short questionnaires with a total of 15 questions. By 'thinking aloud' we mean we would like you to talk us through what you are thinking as you answer the questions. We will record the interview on tape to make sure your views are accurately reported. The interviewer will explain about 'thinking aloud' more fully at the start of the interview, and then have a chat with you at the end. We expect the interview to last no longer than 45 minutes. You are welcome to take breaks during the interview as and when you see fit.

What are the possible benefits of taking part?
Your views as a patient will provide important research input. You will help us understand if the questions asked in quality of life questionnaires are easy to understand and if the questions are important to your quality of life. You will also have a chance to discuss parts of quality of life that you do not think are being measured by these questionnaires.

Are there any disadvantages?
There is a small chance that you may find some of the research questions difficult to answer and upsetting to talk about your quality of life. You are free to withdraw from the interview at any time. We will destroy the interview recorded, if that is what you want.

Will my taking part in the study be kept confidential?
Yes. We will take great care to protect the confidentiality of the information you give us. If you agree to take part and have the interview recorded, we will use a non-personal code to identify the recording so that you cannot be recognised. Any names or places you mention during the interview will be anonymised. The questionnaire you complete will be identified by a study number. The recorded interview will be securely stored through the University of Bristol Research Data Storage Facility, at which point the tape recorded version on the pin protected digital recording device will be destroyed. Only members of the research team will have access to any information you provide during the interview.

Mitchell PM, *et al. BMJ Open* 2020; 10:e034569. doi: 10.1136/bmjopen-2019-034569

What if there is a problem?

If you have any concern about any aspect of this study, please feel free to speak to one of the researchers (Dr. Paul Mitchell, Dr. Fergus Caskey, Dr. Jemima Scott or Professor Joanna Coast, who can be contacted through the School of Social and Community Medicine, University of Bristol, Canynge Hall, 39 Whatley Road, Bristol, BS8 2PS). If you wish to complain formally, you can send a written complaint to Patient advice and liaison services (PALS): Southmead Hospital and address it to Southmead Road, Westbury-on-Trym, Bristol, Avon, BS10 5NB.

What will happen to the results of the research study?

The results will be written up for researchers in medicine and the social sciences. The results will be useful in deciding what quality of life questionnaires to use when evaluating kidney care treatments. In deciding how to measure quality of life, we think it is important to ask people themselves what they think and not just rely on the views of professionals or the government.

Please be aware that all data we collect from this study will be retained for ten years, in accordance with standard University of Bristol data management practice. Anonymised quotations from this interview may also be used in study reports and publications.

Who is organising and funding the research?

This study is being carried out by the University of Bristol in conjunction with the Richard Bright Renal Unit, Southmead Hospital Bristol. The research team comprises three members. Dr Paul Mitchell is the lead researcher for this project. Dr Fergus Caskey is the Medical Director of the UK Renal Registry, a Consultant Nephrologist and a Senior Clinical Lecturer at the School of Social and Community Medicine, University of Bristol. Dr Jemima Scott is an Academic Clinical Fellow in renal care at Southmead Hospital Bristol. Professor Joanna Coast is a Professor in the Economics of Health and Care at the School of Social Science and Community Medicine, University of Bristol.

The research is funded through Paul's work with the UK Renal Registry who are based at Southmead Hospital Bristol and an organisation funded by the NHS to do health and care research called the NIHR CLAHRC West.

Mitchell PM, *et al. BMJ Open* 2020; 10:e034569. doi: 10.1136/bmjopen-2019-034569

Who has reviewed the study?
All research in the NHS is looked at by an independent group of people, called a Research Ethics Committee, to protect your interests. This study has been reviewed and given a favourable opinion by the East of England – Cambridge Central Research Ethics Committee (REF: 16/EE/0331).

If you decide to participate you will have a copy of this information sheet and a signed consent form to keep.

Further information and contact details
Specific information about this research can be provided by lead researcher Dr. Paul Mitchell (e-mail: paul.mitchall@bristol.ac.uk or telephone number 0117 342 1264).

If you would like to receive a copy of the results of this study, please contact Paul using the contact details above. We expect the results of this study to be published by the end of 2018.

Following the interview you may find there are issues that have been discussed about which you would like further support. If this is the case, you can talk to staff in the renal unit where further services can be offered. Or you may find the following national resources helpful:

British Kidney Patient Association: www.britishkidney-pa.co.uk

Think Kidneys National Programme: www.thinkkidneys.nhs.uk

Mitchell PM, et al. BMJ Open 2020; 10:e034569. doi: 10.1136/bmjopen-2019-034569
