# Supplementary material for: Response process validity of three patient reported outcome measures for people requiring kidney care: a think-aloud study using the EQ-5D-5L, ICECAP-A and ICECAP-O
Source: BMJ Open. 2020 May 15;10(5):e034569. doi: 10.1136/bmjopen-2019-034569 (PMC7232621; doi:10.1136/bmjopen-2019-034569)
Supplement: Supplementary data [file bmjopen-2019-034569supp001.pdf]

School of Social and Community Medicine
NIHR CLAHRC West, 9th Floor Whitefriars
BRISTOL BS1 2NT
United Kingdom

+44.117.3421264
paul.mitchell@bristol.ac.uk
www.bristol.ac.uk/social-community-medicine/people/paul-m-mitchell/index.html

STUDY PROTOCOL

Kidney patients' views on quality of life questionnaires: a 'think-aloud' study

Paul M. Mitchell, Fergus J. Caskey, Joanna Coast

Background Information

Since the establishment of the National Institute for Health and Care Excellence (NICE) in the UK in 1999, all new health technologies and clinical guidelines developed for the NHS are required to be assessed for cost-effectiveness. Quality-adjusted life years (QALYs), that combine both health related quality of life and life years into a single metric, is the standard outcome measure in economic evaluations. Generic health related quality of life is recommended to be measured using short self-complete questionnaires, with the EQ-5D measure recommended by NICE (NICE, 2014).

As NICE's remit continues to expand into broader areas such as public health and social care, there is increasing interest in looking at ways of incorporating additional information on patient benefit into cost-effectiveness analysis. There is increasing interest among health economists to measure outcomes from health and related interventions that assess broader wellbeing, allowing for cross-sectoral comparisons across health care and other public bodies (Brazier and Tsuchiya, 2015). One such approach in measuring broader wellbeing has focused on individual's capability to do and be the things in life that matter to them, as an alternative to focusing solely on

Mitchell PM, *et al. BMJ Open* 2020; 10:e034569. doi: 10.1136/bmjopen-2019-034569

health status. Capability measures have been developed for the assessment of specific aspects of health and care, such as chronic pain (Kinghorn et al. 2015), public health (Lorgelly et al. 2015), mental health (Simon et al. 2013) and social care (Netten et al. 2012).

Another approach in measuring capability directly has been to develop short generic measures of perceived capability that could be applied across patient groups receiving health and social care interventions, but targeted to capture capability at different stages of life. The ICEpop CAPability measures, or ICECAP, attempt to capture the capability of all adults aged 18 years and older on the ICECAP-A (Al-Janabi et al. 2012), older adults who are aged 65 years and older on the ICECAP-O (Grewal et al. 2006, Coast et al. 2008) and more recently, a measure for those near the end of life, known as the supportive care measure or ICECAP-SCM (Sutton & Coast, 2014). Whereas the ICECAP-SCM has been designed specifically for programmes towards the end of life, the ICECAP-A and ICECAP-O allow for broader comparisons across health and social care interventions.

Both the ICECAP-A and ICECAP-O have five attributes of capability wellbeing (that is, wellbeing in terms of what people can "do" and "be") with three directly comparable attributes concerning "attachment", "enjoyment" and "autonomy (A)"/ "control (O)" and two less comparable attributes, albeit with overlapping themes: "achievement (A)" and "role (O)", and "stability (A)" and "security (O)" (see Table 1). Both instruments were developed using a similar qualitative interview process, where participants from the general public were asked to specify the aspects of quality of life that were of primary importance to them (Grewal et al. 2006, Al-Janabi et al. 2012). The descriptive system for both measures has four levels in each attribute, ranging from high to no capability, meaning 1024 (4^5) capability states are captured on both measures. Questions are phrased to capture a person's ability to achieve by asking whether an individual "can" or "is able" to achieve in different domains. Both the ICECAP-A and ICECAP-O are conceptually different from generic measures of health functioning, like the EQ-5D, commonly used in health economics (Davis et al. 2013, Keeley et al. 2016). The choice of self-complete questionnaire could also have important resource allocation implications, as recent

Mitchell PM, *et al. BMJ Open* 2020; 10:e034569. doi: 10.1136/bmjopen-2019-034569

research suggests those with severe conditions and with depression are likely to receive greater priority when focusing on capability wellbeing (ICECAP-A) compared to health status (EQ-5D-5L) (Mitchell et al. 2015).

NICE have recently added the use of capability measures to their economic evaluation reference case concerning social care (NICE, 2014), and more recently, ICECAP measures have also been recommended for the economic assessment of interventions for long-term conditions in the Netherlands (Zorginstituut Nederland, 2015). Even though the ICECAP-A and ICECAP-O have overlapping themes, it is unclear as to what measure should be used to assess capability for patients requiring social care or living with a long-term health condition. Validity of both measures has taken place using qualitative and quantitative methods, but the comparative performance of both capability measures has not taken place.

Qualitative research validating the ICECAP-A has so far focused on members of the general population (Al-Janabi et al. 2013), and research professionals (Keeley et al. 2013). Some qualitative research has been conducted with the ICECAP-O in patient groups (Horwood et al. 2014, van Leeuwen et al. 2015). All four of these studies have used cognitive interview methods known as 'think aloud', whereby individuals are asked to verbalise their thought process when they are completing the measure (Willis, 2005). This process is thought to give a more realistic picture of the problems individuals face when completing questionnaires, than more probing interview methods that interrupt the task completion (Kuusela & Paul, 2000). Individuals are asked to verbalise their thought process as they complete the questionnaire, to examine the problems individuals encounter, in terms of comprehension, retrieval, judgement and response difficulties. The interviewer will remain silent throughout this process, so long as individuals continue to think out loud.

Mitchell PM, *et al. BMJ Open* 2020; 10:e034569. doi: 10.1136/bmjopen-2019-034569

Table 1. Generic ICECAP measures: attributes and item descriptions

ICECAP-A (Al-Janabi et al. 2012)	ICECAP-O (Coast et al. 2008)
Stability	Attachment*
- an ability to feel settled and secure	- love and friendship
Attachment	Security
- an ability to have love, friendship and support	- thinking about the future without concern
Autonomy	Role
- an ability to be independent	- doing things that make you feel valued
Achievement	Enjoyment*
- an ability to achieve and progress in life	- enjoyment and pleasure
Enjoyment	Control
- an ability to experience enjoyment and pleasure	- an ability to be independent

*The autonomy (A) and control (O) attributes are phrased exactly the same on both measures. Attachment and enjoyment feature on both measures, but are phrased slightly differently. Stability (A) and security (O), and achievement (A) and role (O) are phrased differently in the descriptive system but have some overlapping themes in the qualitative analysis developing both measures.

The aim of this research is to explore the appropriateness of the ICECAP measures in people who require treatment for chronic kidney disease. In health economic analysis, there is interest in a common measure being used where appropriate, so that results across different patient populations have comparable outcomes when assessing cost-effectiveness. Although NICE has recently recommended the use of the ICECAP-O when assessing social care interventions (NICE, 2014), given that the ICECAP-A captures capability across a broader age range, an argument could be made for using the ICECAP-A for this reason. Patients with chronic kidney disease are likely to crossover the age range where both the ICECAP-A (18+) and ICECAP-O (65+) could feasibly be used. This study will therefore assess the appropriateness of each of the ICECAP measures in this patient population, based on the findings from the qualitative interviews.

Mitchell PM, *et al. BMJ Open* 2020; 10:e034569. doi: 10.1136/bmjopen-2019-034569

It is also worth exploring how the completion of the ICECAP measures compare to the EQ-5D-5L, an expanded version (from three to five levels) of the recommended measure for generating QALYs by NICE. Each of these measures could be used in economic evaluations for patients with chronic kidney disease, so comparative information will be useful in this area. Qualitative interviews will allow for such comparisons, both based on the ease of completion of the measures by the patients in the think aloud exercise and also follow-up questioning on how each of the measures account for the patient's perspective of what aspects of quality of life are most important to them.

Study Objective

The objectives of this study are:

- To assess the feasibility of completing the ICECAP measures and the EQ-5D-5L for people receiving treatment for chronic kidney disease.
- To explore the difficulties in completing the three measures in terms of errors in terms of comprehending, retrieving, judging, responding and struggles (i.e. difficulty answering question, but eventually responded appropriately).
- To seek patient views as to how well the different questionnaires capture their quality of life.

Study Site

Patients will be recruited through the Richard Bright Renal Unit, Southmead Hospital Bristol.

Subjects and Recruitment

Inclusion criteria

To be included in the study, patients must meet *all* of the following criteria:

- Have chronic kidney disease (CKD stage 1-5)
- Willing and able to provide informed consent to participate
- Able to communicate in English

Mitchell PM, *et al. BMJ Open* 2020; 10:e034569. doi: 10.1136/bmjopen-2019-034569

Exclusion criteria

To be excluded in the study, patients must meet *any* of the following criteria:

- Do not have chronic kidney disease (CKD stage 1-5)
- Is not willing and able to provide informed consent to participate
- Is not able to communicate in English

Ethical considerations and informed consent

Patients will be directly recruited through the Richard Bright Renal Unit, Southmead Hospital Bristol. A health care professional from the renal unit will identify patients meeting the inclusion criteria and determine whether the patient might wish to participate. Participants will be given the option of completing the interview in a private room at the renal unit or at their home at a time that is convenient for them. At the point of taking informed consent, the researcher (PM) will go through the information sheet with the participant, answer any questions and request informed consent, with this process expected to take approximately 10 minutes.

Sample size determination

Participants will be sampled using purposive sampling, with diversity sought in terms of age (<65 or >65), sex, and type of kidney care received. The study will aim to recruit approximately 25 patients to participate or until data saturation is reached, whereby no new themes are emerging from the interviews. There is no clear sample size for cognitive interviewing. Previous published think aloud studies using ICECAP measures have had sample sizes ranging from 10 (van Leeuwen et al. 2015) to 34 (Al-Janabi et al. 2013) participants. It is anticipated that a sample size of 25 should be adequate to enable the scoring of struggles and errors in the think aloud task, as well as identifying important themes from the interviews and conclusions about the use of the three quality of life measures for patients with chronic kidney disease.

Withdrawal of participants

Participants are free to withdraw from the study at any time. Clinical care *will not* be affected should the participant decide to withdraw from the study.

Mitchell PM, *et al. BMJ Open* 2020; 10:e034569. doi: 10.1136/bmjopen-2019-034569

Study Design

Patients will be interviewed in a private room at the renal unit or in their home. The interview will commence with a recap of the study aims and an explanation of the format of the interview. Participants will be asked a number of questions concerning socio-demographic information such as age, sex, living alone, condition severity and if patients receive dialysis or not.

To get participants warmed-up, a simple think aloud task will be asked in relation to the number of windows an individual has in their house. Then the Global Quality of Life scale (Hyland & Sodergren 1996) will be presented to them as a practice of self-reporting and thinking out loud. Following the completion of the warm-up task, patients will be randomly allocated the ICECAP-A or the ICECAP-O first or last, with the EQ-5D-5L and EQ-VAS completed in between. Participants will not be interrupted unless they are silent for longer than 10 seconds when they will be asked to “keep thinking aloud”.

Following the completion of the three measures, a discussion between the researcher and participant will follow to clarify the informants’ thoughts whilst completing the measures. In particular, attention will be paid to difficulty in answering the different aspects of the measures and where there was judged to have been uncertainty in the response given by the participant.

The interview will conclude with a semi-structured interview format where patients will be asked about their views on the patient reported outcome measures they reported. Namely, interest will be given to measures they felt best captured their quality of life, what they liked about the measures and what aspects of their quality of life did they feel was missing from the questions being asked.

As the completion of self-reported measures of quality of life using the think aloud process can be emotional for the participants as they reflect on their quality of life, it can be a challenging experience for them. Participants will be offered breaks in the interview process if they are overwhelmed by emotions throughout the interview and will be given the option to stop the interview if that is what they would prefer.

Analysis Plan

All interviews will be transcribed verbatim and, from the transcript, three independent raters will code the transcripts with the aim of identifying four types of response problems to the measures, as well as any areas of 'struggle' (i.e. difficulty in answering that is not so severe as to constitute a response problem). Transcripts will be segmented to material relating to each of the attributes on the three measures. The four types of response problems that will be considered are: comprehension, retrieval, judgement and response. A standardised classification scheme will be employed to consistently identify four types of response problems. The classification is based on the survey response model, developed in cognitive psychology, that suggests that participants perform four actions when answering a question item with errors possible at each stage (Tourangeau et al. 2000). To appropriately answer a question using the survey response model, an individual must: (i) understand (comprehend) the question in the way that the researcher intended; (ii) successfully retrieve the appropriate information to answer the question from their long-term memory; (iii) correctly judge how the recalled information should be used to answer the question; and (iv) format the information into a valid response for the questionnaire.

Three raters (PM, FC and JC) will then independently code the 15 segments (5 items per measure) in each transcript as either: (a) error-free, (b) containing one or more errors or (c) as a 'struggle'. The struggle category is used to identify segments where the participant clearly has difficulty answering the question, but eventually reaches an appropriate answer. Consistency between raters on the coding of the data will be assessed using raw agreement and kappa statistics (Cohen, 1960). Following the independent coding, segments will be judged as errors (or struggles) if a majority of coders note a specific type of error (or struggle). Segments where two raters note a struggle or error but disagree on error type, will be discussed, with a code agreed upon by all raters.

Constant comparative methods will be used to derive explanatory themes from the interviews (Strauss & Corbin, 1990). Transcripts will be read and re-read, and categories and sub-categories will be developed to describe emerging themes. Descriptive accounts will be formed, and matrices used to aid comparison. Issues that are likely to be of interest include the *nature* of response problems across the

Mitchell PM, *et al. BMJ Open* 2020; 10:e034569. doi: 10.1136/bmjopen-2019-034569

different measures, as well as the number of struggles and errors noted for the EQ-5D-5L compared to the ICECAP-A and the ICECAP-O. Themes will focus around reasoning behind preferred measures, aspects of measures they did not like and aspects of quality of life they felt were missing from all measures. Any other themes that arise during completion of the questionnaire and subsequent interview will also be examined.

Data Management

Confidentiality

Confidentiality of all information will be maintained in line with the Data Protection Act. Names and addresses of informants will not be linked to the data obtained and individuals will be identified on transcripts by means of a serial number only. Reporting of data will be in the form of anonymised quotes. Individuals will never be identified in person. Names and addresses of participants will not be released to any outside body or organisation.

Source documents

Interviews will be audio recorded and transcribed verbatim.

Records retention

Research data will include audio-tapes and computer files. Transcripts will be made of audio-tapes, at which point they will be anonymised and all identifiers and potential identifiers removed. Tapes will be kept in a locked filling cabinet and destroyed following the completion of transcribing and primary analysis of the interviews.

Sponsorship and ethical arrangements

Sponsorship of this research project is provided by the University of Bristol (study 2650). Ethics is sought from the NHS Research Ethics Committee.

Insurance

Liability insurance cover for this study is provided by the University of Bristol.

Publication Policy

This research will be written up for peer reviewed publication and submitted to a relevant journal, such as *Quality of Life Research, Social Science & Medicine* or *Value in Health* or a relevant renal journal interested in qualitative research and/or the measurement of quality of life. This study aims to provide valuable research information for a larger research fellowship proposal concerning the use of multiple outcomes in economic evaluations and how it can aid decision-making, with a case study developed in patients with end stage renal disease.

Study Personnel

Paul M. Mitchell, PhD, is a Senior Research Associate at the School of Social and Community Medicine, University of Bristol. Paul is currently funded through a postdoctoral research fellowship in health economics, jointly awarded by the UK Renal Registry and NIHR CLAHRC West.

Fergus J. Caskey, MBChB, MSc, MD, FRCP, is a Senior Clinical Lecturer at the School of Social and Community Medicine, University of Bristol, and Medical Director of the UK Renal Registry, Southmead Hospital Bristol. Dr Caskey is a consultant nephrologist who has vast experience of conducting research within the kidney patient population.

Joanna Coast, PhD, is a Professor of the Economics of Health and Care at the School of Social and Community Medicine, University of Bristol. Professor Coast has particular expertise in the application of qualitative methods, including think aloud studies, in health economics. She was the lead developer for the ICECAP capability measures.

Conflicts of interest

Joanna Coast was the lead developer for the ICECAP measures. Paul Mitchell and Fergus Caskey have no conflicts of interests to declare.

Mitchell PM, *et al. BMJ Open* 2020; 10:e034569. doi: 10.1136/bmjopen-2019-034569

References

Al-Janabi, H., Flynn, T. N., & Coast, J. (2012). Development of a self-report measure of capability wellbeing for adults: the ICECAP-A. *Quality of Life Research*, 21, 167-176.
Al-Janabi, H., Keeley, T., Mitchell, P., & Coast, J. (2013). Can capabilities be self-reported? A think aloud study. *Social Science & Medicine*, 87, 116-122.
Brazier, J., & Tsuchiya, A. (2015). Improving Cross-Sector Comparisons: Going Beyond the Health-Related QALY. *Applied Health Economics and Health Policy*, 13, 557-565.
Coast, J., Flynn, T.N., Natarajan, L., Sproston, K., Lewis, J., Louviere, J.J., & Peters, T.J. (2008). Valuing the ICECAP capability index for older people. *Social Science & Medicine*, 67, 874-882.
Cohen, J. (1960). *A coefficient of agreement for nominal scales*. Educational and Psychological Measurement, 20, 37-46.
Davis, J.C., Liu-Ambrose, T., Richardson, C.G., & Byran S. (2013). A comparison of the ICECAP-O with EQ-5D in a falls prevention clinical setting: are they complements or substitutes? *Quality of Life Research*, 22, 969-977.
Grewal, I., Lewis, J., Flynn, T., Brown, J., Bond, J., & Coast, J. (2006). Developing attributes for a generic quality of life measure for older people: Preferences or capabilities? *Social Science & Medicine*, 62, 1891-1901.
Horwood, J., Sutton, E., & Coast, J. (2014). Evaluating the Face Validity of the ICECAP-O Capabilities Measure: A "Think Aloud" Study with Hip and Knee Arthoplasty Patients. *Applied Research in Quality of Life*, 9, 667-682.
Hyland, M.E., & Sodergren, S.C. (1996) Development of a new type of global quality of life scale, and comparison of performance and preference for 12 global scales. *Quality of Life Research*, 5, 469-480.
Keeley, T., Al-Janabi, H., Lorgelly, P., & Coast, J. (2013). A Qualitative Assessment of the Content Validity of the ICECAP-A and the EQ-5D-5L and Their Appropriateness for Use in Health
Keeley, T., Coast, J., Nicholls, E., Foster, N.E., Jowett, S., & Al-Janabi, H. (2016). An analysis of the complementarity of ICECAP-A and EQ-5D-3L in an adult population of patients with knee pain. *Health and Quality of Life Outcomes*, 14, 36.
Kinghorn, P., Robinson, A., & Smith, R.D. (2015). Developing a Capability-Based Questionnaire for Assessing Well-Being in Patients with Chronic Pain. *Social Indicators Research*, 120, 897-916.
Lorgelly, P., Lorimer, K., Fenwick, E.A.L., Briggs, A.H., & Anand, P. (2015). Operationalising the capability approach as an outcome measure in public health: The development of the OCAP-18. *Social Science & Medicine*, 142, 68-81.
Mitchell, P.M., Al-Janabi, H., Richardson, J., Iezzi, A., & Coast, J. (2015) The relative impacts of disease on health status and capability wellbeing: a multi-country study. *PLoS ONE*, 10, e0143590.
NICE (2014). Developing NICE guidelines: the manual. National Institute for Health and Care Excellence, London, UK, 123.
Netten, A., Burge, P., Malley, J., Potoglou, D., Towers, A-M., Brazier, J., Flynn, T., Forder, J., & Wall B. (2012). Outcomes of social care for adults: developing a preference-weighted measure. *Health Technology Assessment*, 16, 1-165.
Simon, J., Anand, P., Gray, A., Rugkåsa, J., Yeeles, K., & Burns T. (2013). Operationalising the capability approach for outcome measurement in mental health research. *Social Science & Medicine*, 98, 187-196.
Strauss, A., & Corbin, J. (1990). *Basics of qualitative research*. California, US: Sage Publications.

Mitchell PM, *et al. BMJ Open* 2020; 10:e034569. doi: 10.1136/bmjopen-2019-034569

Sutton, E.J., & Coast, J. (2014). Development of a supportive care measure for economic evaluation of end-of-life care using qualitative methods. *Palliative Medicine*, 28, 151-157.
Tourangeau, R., Rips, L., & Rasinski, K. (2000). *The psychology of survey response*. New York: Cambridge University Press.
van Leeuwen, K.M., Jansen, A.P.D., Muntinga, M.E., Bosmans, J.E., Westerman, M.J., van Tulder, M.W., & van der Horst, H.E. (2015). Exploration of the content validity and feasibility of the EQ-5D-3L, ICECAP-O and ASCOT in older adults. *Value in Health*, 15, 201.
Willis, G. (2005). *Cognitive interviewing: A tool for improving questionnaire design*. Thousand Oaks, CA: Sage Publications.
Zorginstituut Nederland (2015). Richtlijn voor het uitvoeren van economische evaluaties in de gezondheidszorg. Web link: https://www.zorginstituutnederland.nl/binaries/content/documents/zinl-www/pakket/werkwijze-pakketbeheer/beoordeling-geneesmiddelen/farmaco-economisch-onderzoek/farmaco-economisch-onderzoek/farmaco-economisch-onderzoek/zinl%3Adocuments/1511-richtlijn-voor-het-uitvoeren-van-economische-evaluaties-in-de-gezondheidszorg/Richtlijn+voor+het+uitvoeren+van+economische+evaluaties+in+de+gezondheidszorg.pdf

Mitchell PM, *et al. BMJ Open* 2020; 10:e034569. doi: 10.1136/bmjopen-2019-034569
