# Supplementary material for: Response process validity of three patient reported outcome measures for people requiring kidney care: a think-aloud study using the EQ-5D-5L, ICECAP-A and ICECAP-O
Source: BMJ Open. 2020 May 15;10(5):e034569. doi: 10.1136/bmjopen-2019-034569 (PMC7232621; doi:10.1136/bmjopen-2019-034569)
Supplement: Author's manuscript [file bmjopen-2019-034569.draft_revisions.pdf]

BMJ Open

BMJ Open is committed to open peer review. As part of this commitment we make the peer review history of every article we publish publicly available.

When an article is published we post the peer reviewers' comments and the authors' responses online. We also post the versions of the paper that were used during peer review. These are the versions that the peer review comments apply to.

The versions of the paper that follow are the versions that were submitted during the peer review process. They are not the versions of record or the final published versions. They should not be cited or distributed as the published version of this manuscript.

BMJ Open is an open access journal and the full, final, typeset and author-corrected version of record of the manuscript is available on our site with no access controls, subscription charges or pay-per-view fees (http://bmjopen.bmj.com).

If you have any questions on BMJ Open's open peer review process please email info.bmjopen@bmj.com

BMJ Open

The face validity and appropriateness of three patient reported outcome measures for people requiring kidney care: a think-aloud study using the EQ-5D-5L, ICECAP-A and ICECAP-O

Journal:	*BMJ Open*
Manuscript ID	bmjopen-2019-034569
Article Type:	Original research
Date Submitted by the Author:	26-Sep-2019

Complete List of Authors:	Mitchell , Paul; University of Bristol, Health Economics Bristol<br>Caskey, Fergus; University of Bristol, Population Health Sciences, University of Bristol; UK Renal Registry, Southmead Hospital Bristol<br>Scott, Jemima; UK Renal Registry, Southmead Hospital Bristol; Southmead Hospital, North Bristol NHS Trust<br>Sanghera, Sabina ; University of Bristol, Health Economics Bristol, Population Health Sciences<br>Coast, Joanna; University of Bristol, Health Economics Bristol, Population Health Sciences

Keywords:	HEALTH ECONOMICS, capability approach, QUALITATIVE RESEARCH, quality of life, wellbeing, kidney care

SCHOLARONE™
Manuscripts

I, the Submitting Author has the right to grant and does grant on behalf of all authors of the Work (as defined in the below author licence), an exclusive licence and/or a non-exclusive licence for contributions from authors who are: i) UK Crown employees; ii) where BMJ has agreed a CC-BY licence shall apply, and/or iii) in accordance with the terms applicable for US Federal Government officers or employees acting as part of their official duties; on a worldwide, perpetual, irrevocable, royalty-free basis to BMJ Publishing Group Ltd ("BMJ") its licensees and where the relevant Journal is co-owned by BMJ to the co-owners of the Journal, to publish the Work in this journal and any other BMJ products and to exploit all rights, as set out in our licence.

The Submitting Author accepts and understands that any supply made under these terms is made by BMJ to the Submitting Author unless you are acting as an employee on behalf of your employer or a postgraduate student of an affiliated institution which is paying any applicable article publishing charge ("APC") for Open Access articles. Where the Submitting Author wishes to make the Work available on an Open Access basis (and intends to pay the relevant APC), the terms of reuse of such Open Access shall be governed by a Creative Commons licence – details of these licences and which Creative Commons licence will apply to this Work are set out in our licence referred to above.

Other than as permitted in any relevant BMJ Author's Self Archiving Policies, I confirm this Work has not been accepted for publication elsewhere, is not being considered for publication elsewhere and does not duplicate material already published. I confirm all authors consent to publication of this Work and authorise the granting of this licence.

For peer review only

The face validity and appropriateness of three patient reported outcome measures for people requiring kidney care: a think-aloud study using the EQ-5D-5L, ICECAP-A and ICECAP-O

Mitchell, Paul Mark,[1*] Caskey, Fergus John,[2-4] Scott, Jemima,[3-4] Sanghera, Sabina,[1] Coast, Joanna[1]

Affiliations

1. Health Economics Bristol (HEB), Population Health Sciences, University of Bristol, Bristol, UK.
2. Population Health Sciences, University of Bristol, Bristol, UK
3. UK Renal Registry, Southmead Hospital Bristol, Bristol, UK.
4. Southmead Hospital Bristol, North Bristol NHS Trust, Bristol, UK

*** Corresponding author:**

E-mail: paul.mitchell@bristol.ac.uk

Phone: +44 (0)117 428 3122

Postal address: 1-5 Whiteladies Road (Room 2.05), Bristol, BS8 1NU, UK

Keywords: health economics; capability approach; qualitative research; quality of life; wellbeing

Word count – excluding title page, references, figures and tables: 3,996

For peer review only - http://bmjopen.bmj.com/site/about/guidelines.xhtml

Abstract

Objectives: To determine the face validity, feasibility of completion, acceptability and preferences for three patient reported outcome measures that could be used in economic evaluation - the EQ-5D-5L, ICECAP-A and ICECAP-O in people requiring kidney care.

Design: Participants were asked to 'think-aloud' while completing the EQ-5D-5L, ICECAP-A and ICECAP-O, followed by a semi-structured interview. Five raters identified errors or struggles in completing the measures from the think-aloud component of the transcripts. Patient preferences for measures were extracted from the semi-structured interview.

Setting: Eligible patients were identified through a large UK secondary care renal centre.

Participants: In total, 30 participants were included in the study, consisting of patients attending renal outpatients for chronic kidney disease (n=18), with a functioning kidney transplant (n=6), and receiving haemodialysis (n=6).

Results: Participants had few errors and struggles in completing the EQ-5D-5L (11% error rate, 3% struggle rate), ICECAP-A (2% error rate, 2% struggle rate) and ICECAP-O (4% error rate, 3% struggle rate). The main errors with the EQ-5D-5L were judgements that did not comply with the "your health today" instruction. Comprehension errors were most prominent on ICECAP-O. Judgement errors were the only errors reported on ICECAP-A. Although the EQ-5D-5L had slightly more errors and struggles, it was the measure most preferred, with participants able to make a clearer link with EQ-5D-5L and their health condition.

Conclusions: The EQ-5D-5L, ICECAP-A and ICECAP-O are feasible for people requiring kidney care to complete and can be included in studies conducting economic evaluations of kidney care interventions. Further research is required to assess how health (e.g. EQ-5D) and capability (e.g. ICECAP) measures can be included in an economic evaluation simultaneously, as well as what ICECAP measure(s) to include when patient groups straddle the age ranges for ICECAP-A (18 years and older) and ICECAP-O (65 years and older).

Strengths and limitations of this study

- This is the first study to include EQ-5D-5L, ICECAP-A and ICECAP-O in a think-aloud study with people requiring kidney care
- The sample consists of a broad range of people requiring kidney care including renal outpatients for chronic kidney disease, kidney transplant check-ups and haemodialysis
- Think-aloud studies aim to identify errors and struggles in task completion as they occur
- Five raters, with diverse experience across health economics, qualitative research and kidney care, identified errors and struggles from the think-aloud transcripts
- Think-aloud relies on participants verbalising their difficulty in task completion

For peer review only - http://bmjopen.bmj.com/site/about/guidelines.xhtml

BACKGROUND

Healthcare expenditure is rising globally and has been increasing at a faster rate than international economic growth over the past decade.[1] Chronic kidney disease (CKD) is a growing burden on healthcare resources. In the 2015 Global Burden of Disease Study,[2] CKD were the twelfth leading cause of death and seventeenth leading cause of global life years lost.[3] In the UK alone, CKD accounts for more than one per cent of the national health service (NHS) annual budget.[4] Given this volume of expenditure, it is important that any healthcare resources allocated to managing kidney problems are used efficiently.

To determine which interventions should be recommended for practice, economic evaluations provide evidence on cost-effectiveness by comparing the costs and benefits of alternative interventions. In health and care, these economic evaluations increasingly rely on patient reported outcome measures (PROMs) to capture the health-related quality of life improvements from interventions[5] and are recommended for the generation of quality adjusted life years (QALYs) internationally.[6] A QALY is a combination of life years adjusted for health related quality of life.[7] Choice of PROM in generating QALYs plays an influential role in deciding if a treatment is cost-effective.[8 9] The EQ-5D is the most widely used measure to calculate QALYs in economic evaluations internationally [5] and has been translated into 169 different languages.[10] The EQ-5D has also been separately recommended by an expert consensus for routine collection across European renal registries.[11]

Despite international recommended use of QALYs in healthcare, the suitability of this outcome is debated, partly due to the exclusive focus on health gains and not broader wellbeing.[7 12 13] An alternative approach has been proposed to capture broader wellbeing, which focuses on a person's capabilities, meaning a person's freedom to achieve the things in life that are valuable to them.[14] Health bodies in the UK and the Netherlands have recognised the limitation of relying purely on QALYs in social care[15] and long-term health conditions.[16] Capability measures, such as the ICECAP-A[17] (A- all adults aged 18 years and above) and the ICECAP-O (O- older adults aged 65 years and above),[18] have been recommended as ways to capture the broader benefits for these patient groups. It is not entirely clear, however, which ICECAP measure to use when the age range of a patient group could use either ICECAP-A or ICECAP-O. People requiring kidney care are a prime example of this challenge, with the median age for starting renal replacement therapy in the UK being 64 years of age in 2017.[19] Although the validity of ICECAP measures in people requiring kidney care is emerging,[20] no previous study has tested the ICECAP-A and ICECAP-O in the same patient group.[21]

The objective of this study was to (1) explore face validity, feasibility of completion and acceptability of the health related quality of life PROM EQ-5D-5L,and two capability PROMs of broader wellbeing,

For peer review only - http://bmjopen.bmj.com/site/about/guidelines.xhtml

ICECAP-A and ICECAP-O, in patients requiring kidney care, and (2) assess patient preferences for the three PROMs.

METHODS

This research consists of a 'think-aloud' study followed by a semi-structured interview. A think-aloud study is a cognitive interview method whereby individuals are asked to verbalise their thought process when completing measures.[22] Think-aloud interviews enabling examination of the problems patients may encounter in terms of comprehension, retrieval, judgement and response difficulties. The interviewer remains silent, so long as individuals continue to think-aloud. This process is thought to give a more realistic picture of the problems that individuals face when completing questionnaires than more direct interview methods that interrupt task completion.[23] Ethics approval was obtained from the East of England NHS Research Ethics Committee (16/EE/0331) (see supplementary file 1 for research protocol).

Sampling and recruitment

Samples for previous think-aloud studies on health and capability PROMs have ranged from 10 people (van Leeuwen et al. 2015) to 34 (Al-Janabi et al. 2013). *A priori*, it was anticipated that a sample size of at least 25 patients would be sufficient to enable the analysis of the think-aloud tasks, as well as further exploring the preferences of the three PROMs (EQ-5D-5L, ICECAP-A and ICECAP-O) for people requiring kidney care.

Patients were recruited through a large UK secondary care renal centre. Participants were sampled purposefully to achieve diversity in age (<65 or >65) and type of kidney care received. Sampling was conducted through renal outpatient lists and a dialysis unit. Eligibility required individuals to have chronic kidney disease, be willing and able to provide informed consent to participate, and be able to communicate in English (because the study was exploring the use of English language questionnaires). Potential participants received a participant information sheet (PIS – see supplementary file 2) in the post or at the dialysis unit and were invited to take part via a follow-up telephone call from a clinical trials officer. The PIS was the only information provided to the participant about the researcher prior to interview.

Instruments investigated

The EQ-5D-5L consists of five dimensions of health status covering mobility, self-care, usual activities, pain/discomfort and anxiety/depression across five levels ranging from no problems to extreme problems.[24] The EQ-5D-5L was introduced to supersede the EQ-5D-3L to reduce ceiling effects and

For peer review only - http://bmjopen.bmj.com/site/about/guidelines.xhtml

increase sensitivity to change. A Visual Analogue Scale (VAS) is also included that asks respondents to rate their health today on a 0-100 scale from worst to best imaginable health state.

The ICECAP-A is a capability wellbeing measure developed for the general adult population.[17] It consists of five dimensions relating to a person's capability to have attachment, stability, achievement, enjoyment and autonomy. Each dimension has four levels ranging from no capability to full capability. The capabilities were identified through qualitative research with members of the general public to identify what was most important to them in their life.[17]

The ICECAP-O is a capability wellbeing measure developed for older adults.[18] The ICECAP-O was the first of the ICECAP suite of measures developed that aimed to develop a more appropriate quality of life measure for older adults specifically for use in the economic evaluation of health and care interventions.[25] It consists of five dimensions relating to a person's capability to have attachment, security, role, enjoyment and control. Each dimension has four levels ranging from no capability to full capability. As with the ICECAP-A, the capabilities were identified through qualitative research, but in this case with older members of the general public aged 65 years and above.[26]

Data collection

Once participants had provided informed consent, interviews took place at the renal centre or in the participant's home. All interviews were conducted by PM, a male PhD researcher in health economics with qualitative interview training and an interest in PROMs research. Initial questions focused on basic socio-demographic information. Participants then completed a simple warm-up task to determine the number of windows in their home. A second warm up task involved the completion of the Global Quality of Life scale.[27] Participants then completed the think-aloud exercise. They were allocated sequentially to receive ICECAP-A or ICECAP-O first or third, with EQ-5D-5L (including the EQ-VAS) always completed second; given the similarities between ICECAP-A and ICECAP-O, it was seen as a stronger design to separate these two measures to avoid confusion. Participants were not interrupted during the completion of the three measures unless they were silent for longer than 10 seconds when they were asked to "keep thinking aloud". Following the think-aloud task, a semi-structured interview was conducted to clarify issues arising in the think-aloud task and to explore views about the three measures. Field notes were made during the think-aloud component to guide the semi-structured interview. The interview guide was piloted prior to interview. Transcripts were not returned to participants for comment and/or correction and they did not provide feedback on the study findings. Repeat interviews were not carried out. Interviews were audio recorded and transcribed verbatim. Data were managed in Microsoft Word and Excel.

For peer review only - http://bmjopen.bmj.com/site/about/guidelines.xhtml

Data analysis

i. Think-aloud analysis

The think-aloud section from each transcript was extracted for each of the measures and divided into 16 segments: 6 representing the items on the EQ-5D-5L (including the EQ-5D-VAS), 5 items on the ICECAP-A and ICECAP-O, respectively. Think-aloud sections of the interview, alongside the reported response level for each item on each measure, were presented to five independent raters (PM, FC, JS, SS and JC), with expertise in health economics (PM, SS and JC), qualitative research (JC) and renal care (FC and JS). Each rater individually examined all think-aloud sections to identify problems participants encountered when completing each of the three measures. Raters were asked to identify whether responses were error free or contained any of the following problems, based on the survey response model [28]:

1. Comprehension error (understanding the question in the way the researcher intended)
2. Retrieval error (retrieving appropriate information from their long-term memory)
3. Judgement error (correctly judging how recalled information should be used to answer)
4. Response error (format the information into a valid response for the questionnaire)
5. Struggle (not one of the four errors but clear difficulty in answering the question) [29]

Following these independent ratings, each item was identified as error free, containing an error or containing a struggle, using the following rules:

- Where three or more raters identified a specific error/struggle it was classed as an error/struggle;
- Where one or none thought an error was present it was marked as error free;
- Where two or more raters identified an error/struggle but there was no majority agreement on the type of error/struggle, a decision was made during a consensus meeting with all raters; a majority decision was used when no consensus occurred.

Consistency between raters on the coding of the data was assessed using raw agreement and a weighted kappa statistic.[30] For the latter, where an error and no error were reported between raters, this was weighted as 0; all other disagreements - such as different error types, error/struggle or struggle/no error - was weighted as 0.5; with agreement weighted as 1.

For peer review only - http://bmjopen.bmj.com/site/about/guidelines.xhtml

ii. Preference between measures

A part of the semi-structured interviews following the think-aloud task explored individual preferences for completing the three measures. Individuals were asked which of the three measures they preferred and why they thought it was more important in assessing their quality of life. Where EQ-5D was the preferred option, a follow-up question was asked about preferences between the two ICECAP measures.

Patient and public involvement

Patients and the public were not directly involved in the design of the study.

RESULTS

334 patients were invited to take part in the study. Of these, 161 responded to telephone follow-up and 37 agreed to participate. In four cases, patients did not attend the interview, one individual was too unwell to participate and one decided against participation during the consent process. Thirty-one individuals took part, but one individual did not understand the task (reading aloud their response levels only), leaving 30 individuals as the final sample. Most interviews took place at the health care facility, with four taking place in the participant's home. Most interviews were conducted one-to-one; on occasion at the health care facility patients' partners were present. Interviews were conducted between April and July 2017 and lasted between 16 and 55 minutes (average 33 minutes). Characteristics of the sample are presented in Table 1.

Think-aloud analysis: Errors and struggles

Following independent coding of the think-aloud interviews by five raters, inter-rater agreement was similar for ICECAP-A (85%-95%) and ICECAP-O (83%-93%), slightly lower for EQ-5D-5L (78%-84%) and weighted chance-corrected agreement being rated 'fair' to 'moderate' for 29 out of 30 inter-rater comparisons using standard guidelines.[31] Eight errors (4 EQ-5D-5L, 0 ICECAP-A and 4 ICECAP-O), eight struggles (5 EQ-5D-5L, 1 ICECAP-A and 2 ICECAP-O) and 52 possible error/struggles were identified through independent rating. At the subsequent rater meeting, from the 52 possible error/struggles, a further 26 errors or struggles were agreed upon: 17/29 for the EQ-5D-5L, 5/11 for the ICECAP-A and 4/12 for the ICECAP-O. Breakdowns of error type by measure item are reported in tables 2-4. In total, 179 segments were generated for the EQ-5D-5L (one VAS was not completed by accident) and 150 segments each for the ICECAP-A and ICECAP-O. Twenty (11%) out of the 179 segments of the EQ-5D-5L were associated with an error and 6 (3%) with a struggle. Three (2%) out of the 150 segments of the ICECAP-A were associated with an error and 3 (2%) with a struggle. Six (4%) out of the 150 segments of the ICECAP-O were associated with an error and 4 (3%) with a struggle.

For peer review only - http://bmjopen.bmj.com/site/about/guidelines.xhtml

Table 1 Participants' characteristics (n=30)

Sex	
Male	23
Female	7
Ethnicity	
White	28
Non-white	2
Age group	
75+	4
65-74	8
55-64	7
45-54	6
35-44	4
18-34	1
Kidney care received	
Renal outpatients	18
Renal outpatients (transplant)	6
Dialysis	6

Table 2 Errors and Struggles: EQ-5D-5L (n=30)

	Mobility	Self-Care	Usual Activities	Pain/ Discomfort	Anxiety/ Depression	VAS*	Total
Error							
Comprehension	0	0	1	0	0	0	1
Retrieval	0	0	0	0	0	0	0
Judgement	1	1	2	6	2	2	14
Response	0	0	2	2	0	1	5
Struggle	1	0	1	2	0	2	6
Total	2	1	6	10	2	5	26

*n=29

Table 3. Errors and Struggles: ICECAP-A (n=30)

	Stability	Attachment	Autonomy	Achievement	Enjoyment	Total
Error						
Comprehension	0	0	0	0	0	0
Retrieval	0	0	0	0	0	0
Judgement	0	0	2	1	0	3
Response	0	0	0	0	0	0
Struggle	2	0	0	1	0	3
Total	2	0	2	2	0	6

Table 4. Errors and Struggles: ICECAP-O (n=30)

	Attachment	Security	Role	Enjoyment	Control	Total
Error						
Comprehension	1	0	2	0	0	3
Retrieval	0	0	0	0	0	0
Judgement	0	0	1	0	0	1
Response	0	0	0	2	0	2
Struggle	1	1	2	0	0	4
Total	2	1	5	2	0	10

For peer review only - http://bmjopen.bmj.com/site/about/guidelines.xhtml

The majority of responses were not identified as an error or struggle on any of the three measures. Participants found all measures easy to complete overall:

"Very straightforward." (Participant 26, male, aged 65-74, dialysis patient)

"Not particularly, they all seemed, they're all pretty relevant to the questionnaire and to my condition and recovery and all that sort of thing so nothing sort of surprised me what was being asked so, happy with all the questions that was fine"

(Participant 21, male, 18-35, kidney transplant outpatient)

There were more errors (17) and struggles (4) reported for EQ-5D-5L (even when excluding the EQ-VAS) than for either ICECAP measure. The most common error type for EQ-5D-5L related to judgment, with this error recorded at least once across all EQ-5D-5L dimensions. Raters decided that a judgement error had occurred when participants clearly diverged from the EQ-5D-5L instruction to focus on "***your health today***":

"I am working – I am doing this on – on a bad day"

(Participant 19, male, aged 55-64, judgement errors for four of five EQ-5D-5L dimensions)

Response errors for the pain/discomfort dimension were driven by the infrequency with which they were reported to occur:

"But sitting here now I would put my state, a little bit of discomfort, but I don't think either end of the spectrum really indicates what I actually feel. Because it is a thing which either comes on and then is put right by antibiotics or painkillers, so I'm going to put moderate pain or discomfort. But perhaps there should be a box for occasional to indicate recurrent or occasional pain." (Participant 12, male, aged 65-74, pain/discomfort response error)

Response errors for usual activities were due to no response being provided and one participant felt that their true response was in between slight and moderate problems. The only other error recorded on the EQ-5D-5L was also for usual activities in terms of comprehension:

"Not sure what my usual activities are. Walking I suppose. I'm sorry I can't think what my usual activities are. So I don't know what to put there."

(Participant 27, male, aged 75+, usual activities response error)

For peer review only - http://bmjopen.bmj.com/site/about/guidelines.xhtml

For the ICECAP-A, there were only judgement errors or struggles reported. The attachment and enjoyment dimensions were error and struggle free. Two of the judgement errors follow a similar pattern as for EQ-5D, where one individual reported their capability on a bad day, rather than at the moment. The other judgement error related to the participants' interpretation of the item:

> *"... I'm reading that one as being completely independent, is that I would probably be quite happy living on my own rather than with a partner or family..."*
>
> (Participant 10, male, aged 65-74, judgement error)

Comprehension errors were the highest error type for ICECAP-O, with two participants unable to understand the role dimension and one participant the attachment attribute:

> *"Question one, love and friendship, reading the supposed answers, I find them rather confusing. I can have all of the love and friendship that I want (-) not really [sure] what the question is asking. Very difficult. Totally bemused by question one, so I will hazard a guess"*
>
> (Participant 8, male, aged 55-64, attachment comprehension error)

> *"I don't want to feel valued. Again I don't understand what this means really. Valued by whom? (-) I don't know I can't answer that at all."*
>
> (Participant 27, male, aged 75+, role comprehension error)

Another error on the role attribute was found when one participant focused on functioning (i.e. what they do) rather than their capability (i.e. what they are able to do):

> *"Yeah, actually, it's interesting if I think about it a bit more actually. I probably am able to do all of the things that make me feel valued but don't actually do them. I think I'll leave that to many of the things"* (Participant 10, male, aged 65-74, role judgement error)

There were two response errors on the enjoyment attribute where both individuals felt they were in between the same two levels:

> *"...don't have all the time that I'd like to spend doing stuff outside the work so my answer's probably a two and a half but I'll put a three."*
>
> (Participant 16, male, aged 55-64, enjoyment response error)

> *"Enjoyment and pleasure? I think I'm somewhere – I'm gonna put myself unhelpfully at two and a half because I don't think I have a little, I don't think I have a lot."*
>
> (Participant 18, male, aged 35-44, enjoyment response error)

For peer review only - http://bmjopen.bmj.com/site/about/guidelines.xhtml

Measure Preferences

Most individuals found it difficult to choose which of the three PROMs was their most preferred measure. Nevertheless, the EQ-5D-5L was most preferred (n=17), five preferred ICECAP-O, three preferred ICECAP-A and five were unable to make a choice. One reason for preferring EQ-5D-5L was that participants could more clearly see the connection between the questions being asked and their illness:

> *"I think the one that…because I've come via the kidney clinic, I'm-I'm thinking that this kidney research rather than general life research, so I think the one that relates most clearly to health and different problems that you might experience with kidney problems… is (EQ-5D-5L)."*
> (Participant 5, female, aged 45-54)

> *"That one about the physical thing. That seemed to be more relevant about whether you're well, ill or what other problems you've got. More relevant for a medical questionnaire rather than how you feel and stuff. But I know how you feel is important as well but you know, whether you can get about and might need help getting to appointments, things like that might be more, more relevant."* (Participant 17, male, aged 35-44)

Reasons for preferring either ICECAP measure were due to what was being measured and a perceived greater depth compared to the EQ-5D-5L:

> *"That one's (EQ-5D-5L) really quite a superficial, can I walk around, can I wash myself, kind of very operational stuff. These two are more about kind of more psychological as well as quite physical things. Other than you talk about anxiety, depression there and I instantly said I don't – clearly they're in my head I'm not depressed, I don't have that illness. I would – so would put down to these two and I would go with this one (ICECAP-A) because I quite like the – the independent, achievement and progress but I think that one was a brilliant question because I think that's probably the most important thing that is on my mind at the moment."*
> (Participant 18, male, aged 35-44)

> *"Because (ICECAP-O) that's… it's all embodying isn't it about your family, your life, what you do, where you think you're going."* (Participant 15, male, aged 65-74)

For peer review only - http://bmjopen.bmj.com/site/about/guidelines.xhtml

DISCUSSION

This study explored the face validity, feasibility of completion and acceptability of EQ-5D-5L, ICECAP-A and ICECAP-O in patients requiring kidney care and preferences between the three PROMs. There were more errors and struggles reported with the EQ-5D-5L, mainly related to judgement errors with respect to the answer provided varying from the measure recall period "*your health today*". Nevertheless, most participants preferred the EQ-5D-5L for reasons of ease of completion and were more directly able to link the wording of the questions to their health condition. ICECAP-A had the fewest errors and struggles overall. One in six participants or more recorded an error or struggle in completing EQ-5D-5L pain/discomfort, EQ-5D-5L usual activities, EQ-5D-5L VAS and ICECAP-O role items.

This study is the first to collect both ICECAP-A and ICECAP-O measures simultaneously in the same patient group. The study benefits from having participants with a broad range of kidney problems and receiving different treatments, as well as including those with a range of different issues. There are some limitations, however: the sample was predominantly male and of white ethnicity. Although most respondents were male, this is not dissimilar to renal replacement therapy recipients in the UK where almost 2 in every 3 patients (64.1%) are male.[19] Nevertheless, the findings need to be interpreted in light of the sample.

As with other similar size studies in different populations, this work has shown that responses to the ICECAP-A measure have fewer errors or struggles than those to EQ-5D. [29 32] It differs from the only existing comparison between EQ-5D-5L and ICECAP-O which, in a smaller study (n=10) found the EQ-5D-5L produced fewer errors in completion.[33] Errors associated with comprehending the attachment and role items on ICECAP-O are similar to previous think-aloud studies.[33 34] A potential concern over the use of the new EQ-5D-5L is the number of judgement errors that were found here. This seems to be particularly related to the prevalence of intermittent health problems for people requiring kidney care, which caused patients difficulty in responding and is mostly related to the timeframe of EQ-5D.

The findings suggest that all three measures are appropriate for use in people requiring kidney care although they have different strengths and weaknesses; the fewer errors reported for the two ICECAP measures may be traded against the patients' preferences for the EQ-5D-5L. Indeed, the finding that the EQ-5D-5L was preferred by patients reflects earlier works showing that patients preferred EQ-5D over a number of other condition-specific and generic measures of health status.[11] From a health and care decision-making point of view, although both errors in completion and patient preferences are important in choice of measure, they are unlikely to be the only considerations for choice of measure to aid in resource allocation decisions across health and care service provision. In a recent review of

For peer review only - http://bmjopen.bmj.com/site/about/guidelines.xhtml

EQ-5D scores (i.e. using population preferences to value the relative importance of health states[7]) attached to health states for calculating QALYs in patients with end stage renal disease (ESRD), there is only a clear benefit attached to the health gain from kidney transplantation compared to other treatments, such as dialysis and conservative care.[35] This finding may not be surprising given health levels for people with kidney transplants have found to be comparable to that of the general population[36] and is generally considered the clinical "gold standard" treatment option for people with ESRD.[37] However, what may be surprising is that the EQ-5D is not able to distinguish patient benefits from the type of dialysis, how dialysis is delivered or whether dialysis is delivered at all. Previous stated preference research from Australia has shown that pre-dialysis patients would be willing to trade-off on average seven months of survival time to reduce the number of trips to hospital for dialysis per week and on average fifteen months of survival time to reduce their restrictions on their ability to travel and make short trips.[38] Such important considerations do not appear to be captured using the current economic toolkit that focus primarily on patient health status and not the impact of that treatment on their broader ability to do and be things in life that matter to them.

Future work could look at how decision-makers can use health and capability measures simultaneously in an economic evaluation. In addition, there are growing critiques of the development of the EQ-5D-5L for use in economic evaluation, in relation to ordering effects[39] and valuation methodology.[9] This study highlights issues surrounding the variation in interpretation and judgements relating to the framing of both EQ-5D versions (i.e. "*your health today*") that are also likely to be of interest to explore further.

Further research is required to better understand whether the different ICECAP measures are completed differently depending on the respondents' stage of life. Measuring capability at different stages across the life course may provide an alternative framework for using the ICECAP capability measures in economic evaluations for health and care interventions.[40] More detailed qualitative analysis of think-aloud and semi-structured interviews may provide some answers in the implementation of such a life course framework.

For peer review only - http://bmjopen.bmj.com/site/about/guidelines.xhtml

Contributorship statement

PM, FC and JC developed the study design. PM, FC and JS were involved in data acquisition. All authors were involved with analysing and interpreting the data. PM initially drafted this paper. All authors were involved in the revision of the initial draft for important intellectual content and final approval of this version to be published. All authors agree to be accountable for all aspects of the work in ensuring that questions related to the accuracy or integrity of any part of the work are appropriately investigated and resolved.

Competing interests

Joanna Coast led the development of the ICECAP-O and ICECAP-A. All other authors have no competing interests to declare.

Funding

This work was initially supported by a post-doctoral fellowship co-funded by the National Institute for Health Research (NIHR) Collaboration for Leadership in Applied Health Research and Care West (NIHR CLAHRC West) and the UK Renal Registry (UKRR). The views expressed in this article are those of the authors and not necessarily those of the NHS, the NIHR, the Department of Health and Social Care or the UKRR. Time for drafting this paper for Paul Mitchell and Joanna Coast has also been supported through a Wellcome Trust investigator award (205384/Z/16/Z).

Data sharing statement

No data are available.

REFERENCES

1. Xu K, Soucat A, Kutzin J, et al. Public Spending on Health: A Closer Look at Global Trends. Switzerland: World Health Organisation 2018.
2. Wang H, Naghavi M, Allen C, et al. Global, regional, and national life expectancy, all-cause mortality, and cause-specific mortality for 249 causes of death, 1980–2015: a systematic analysis for the Global Burden of Disease Study 2015. *The Lancet* 2016;388(10053):1459-544. doi: https://doi.org/10.1016/S0140-6736(16)31012-1
3. Neuen BL, Chadban SJ, Demaio AR, et al. Chronic kidney disease and the global NCDs agenda. 2017;2(2):e000380. doi: 10.1136/bmjgh-2017-000380 %J BMJ Global Health
4. Kerr M, Bray B, Medcalf J, et al. Estimating the financial cost of chronic kidney disease to the NHS in England. *Nephrology Dialysis Transplantation* 2012;27(suppl 3):iii73-iii80. doi: 10.1093/ndt/gfs269
5. Wisløff T, Hagen G, Hamidi V, et al. Estimating QALY Gains in Applied Studies: A Review of Cost-Utility Analyses Published in 2010. *PharmacoEconomics* 2014;32(4):367-75. doi: 10.1007/s40273-014-0136-z

For peer review only - http://bmjopen.bmj.com/site/about/guidelines.xhtml

6. Rowen D, Azzabi Zouraq I, Chevrou-Severac H, et al. International Regulations and Recommendations for Utility Data for Health Technology Assessment. *PharmacoEconomics* 2017;35(1):11-19. doi: 10.1007/s40273-017-0544-y
7. Weinstein MC, Torrance G, McGuire A. QALYs: The Basics. *Value in Health* 2009;12:S5-S9. doi: 10.1111/j.1524-4733.2009.00515.x
8. Richardson J, Khan MA, Iezzi A, et al. Comparing and Explaining Differences in the Magnitude, Content, and Sensitivity of Utilities Predicted by the EQ-5D, SF-6D, HUI 3, 15D, QWB, and AQoL-8D Multiattribute Utility Instruments. *Medical Decision Making* 2014;35(3):276-91. doi: 10.1177/0272989X14543107
9. Hernandez Alava M, Wailoo A, Grimm S, et al. EQ-5D-5L versus EQ-5D-3L: The Impact on Cost Effectiveness in the United Kingdom. *Value in Health* 2018;21(1):49-56. doi: https://doi.org/10.1016/j.jval.2017.09.004
10. Devlin NJ, Krabbe PFM. The development of new research methods for the valuation of EQ-5D-5L. *The European Journal of Health Economics* 2013;14(1):1-3. doi: 10.1007/s10198-013-0502-3
11. Breckenridge K, Bekker HL, Gibbons E, et al. How to routinely collect data on patient-reported outcome and experience measures in renal registries in Europe: an expert consensus meeting. *Nephrology Dialysis Transplantation* 2015;30(10):1605-14. doi: 10.1093/ndt/gfv209
12. Coast J, Smith RD, Lorgelly P. Should the capability approach be applied in Health Economics? *Health Economics* 2008;17(6):667-70. doi: 10.1002/hec.1359
13. Dolan P, Kahneman D. Interpretations Of Utility And Their Implications For The Valuation Of Health*. *The Economic Journal* 2008;118(525):215-34. doi: 10.1111/j.1468-0297.2007.02110.x
14. Sen A. Capability and Well-Being. In: Nussbaum MC, Sen A, eds. The Quality of Life. Oxford, UK: Oxford University Press 1993:30-53.
15. NICE. NICE guidelines: the manual. London: National Institute for Health and Care Excellence, 2018:125.
16. ZorginstituutNederland. Guideline for economic evaluations in healthcare, 2016.
17. Al-Janabi H, Flynn T, Coast J. Development of a self-report measure of capability wellbeing for adults: the ICECAP-A. *Quality of Life Research* 2012;21(1):167-76. doi: 10.1007/s11136-011-9927-2
18. Coast J, Flynn TN, Natarajan L, et al. Valuing the ICECAP capability index for older people. *Social Science & Medicine* 2008;67(5):874-82. doi: http://dx.doi.org/10.1016/j.socscimed.2008.05.015
19. Registry UR. UK Renal Registry 21st Annual Report - data to 31/12/2017. Bristol, UK, 2019.
20. Shah KK, Murtagh FEM, McGeechan K, et al. Health-related quality of life and well-being in people over 75 years of age with end-stage kidney disease managed with dialysis or comprehensive conservative care: a cross-sectional study in the UK and Australia. *BMJ Open* 2019;9(5):e027776. doi: 10.1136/bmjopen-2018-027776
21. Proud L, McLoughlin C, Kinghorn P. ICECAP-O, the current state of play: a systematic review of studies reporting the psychometric properties and use of the instrument over the decade since its publication. *Quality of Life Research* 2019;28(6):1429-39. doi: 10.1007/s11136-019-02114-y
22. Willis GB. Cognitive interviewing: A tool for improving questionnaire design: Sage Publications 2004.
23. Kuusela H, Paul P. A Comparison of Concurrent and Retrospective Verbal Protocol Analysis. *The American Journal of Psychology* 2000;113(3):387-404. doi: 10.2307/1423365
24. Herdman M, Gudex C, Lloyd A, et al. Development and preliminary testing of the new five-level version of EQ-5D (EQ-5D-5L). *Quality of Life Research* 2011;20(10):1727-36. doi: 10.1007/s11136-011-9903-x

For peer review only - http://bmjopen.bmj.com/site/about/guidelines.xhtml

25. Coast J, Kinghorn P, Mitchell P. The Development of Capability Measures in Health Economics: Opportunities, Challenges and Progress. *The Patient - Patient-Centered Outcomes Research* 2015;8(2):119-26. doi: 10.1007/s40271-014-0080-1
26. Grewal I, Lewis J, Flynn T, et al. Developing attributes for a generic quality of life measure for older people: Preferences or capabilities? *Social Science & Medicine* 2006;62(8):1891-901. doi: http://dx.doi.org/10.1016/j.socscimed.2005.08.023
27. Hyland ME, Sodergren SC. Development of a new type of global quality of life scale, and comparison of performance and preference for 12 global scales. *Quality of Life Research* 1996;5(5):469-80. doi: 10.1007/bf00540019
28. Tourangeau R, Rips LJ, Rasinski K. The psychology of survey response: Cambridge University Press 2000.
29. Al-Janabi H, Keeley T, Mitchell P, et al. Can capabilities be self-reported? A think aloud study. *Social Science & Medicine* 2013;87:116-22. doi: http://dx.doi.org/10.1016/j.socscimed.2013.03.035
30. Cohen J. A Coefficient of Agreement for Nominal Scales. *Educational and Psychological Measurement* 1960;20(1):37-46. doi: 10.1177/001316446002000104
31. Altman DG. Practical statistics for medical research. London: Chapman & Hall 1991.
32. Bailey C, Kinghorn P, Orlando R, et al. 'The ICECAP-SCM tells you more about what I'm going through': A think-aloud study measuring quality of life among patients receiving supportive and palliative care. *Palliative Medicine* 2016;30(7):642-52. doi: 10.1177/0269216315624890
33. van Leeuwen KM, Jansen APD, Muntinga ME, et al. Exploration of the content validity and feasibility of the EQ-5D-3L, ICECAP-O and ASCOT in older adults. *BMC Health Services Research* 2015;15(1):201. doi: 10.1186/s12913-015-0862-8
34. Horwood J, Sutton E, Coast J. Evaluating the Face Validity of the ICECAP-O Capabilities Measure: A "Think Aloud" Study with Hip and Knee Arthroplasty Patients. *Applied Research in Quality of Life* 2014;9(3):667-82. doi: 10.1007/s11482-013-9264-4
35. Wyld M, Morton RL, Hayen A, et al. A Systematic Review and Meta-Analysis of Utility-Based Quality of Life in Chronic Kidney Disease Treatments. *PLoS Medicine* 2012;9(9):e1001307. doi: 10.1371/journal.pmed.1001307
36. Villeneuve C, Laroche M-L, Essig M, et al. Evolution and Determinants of Health-Related Quality-of-Life in Kidney Transplant Patients Over the First 3 Years After Transplantation. *Transplantation* 2016;100(3):640-47. doi: 10.1097/tp.0000000000000846
37. Wolfe RA, Ashby VB, Milford EL, et al. Comparison of Mortality in All Patients on Dialysis, Patients on Dialysis Awaiting Transplantation, and Recipients of a First Cadaveric Transplant. *New England Journal of Medicine* 1999;341(23):1725-30. doi: doi:10.1056/NEJM199912023412303
38. Morton RL, Snelling P, Webster AC, et al. Factors influencing patient choice of dialysis versus conservative care to treat end-stage kidney disease. *Canadian Medical Association Journal* 2012;184(5):E277-E83. doi: 10.1503/cmaj.111355
39. Tsuchiya A, Bansback N, Hole A, et al. Manipulating the Five Dimensions of the EuroQol Instrument: The Effects on Self-Reporting Actual Health and Valuing Hypothetical Health States. *Medical Decision Making* 2019
40. Coast J. Assessing capability in economic evaluation: a life course approach? *The European Journal of Health Economics* 2019 doi: 10.1007/s10198-018-1027-6

For peer review only

For peer review only - http://bmjopen.bmj.com/site/about/guidelines.xhtml

School of Social and Community Medicine
NIHR CLAHRC West, 9th Floor Whitefriars
BRISTOL BS1 2NT
United Kingdom

+44.117.3421264
paul.mitchell@bristol.ac.uk
www.bristol.ac.uk/social-community-medicine/people/paul-m-mitchell/index.html

STUDY PROTOCOL

Kidney patients' views on quality of life questionnaires: a 'think-aloud' study

Paul M. Mitchell, Fergus J. Caskey, Joanna Coast

Background Information

Since the establishment of the National Institute for Health and Care Excellence (NICE) in the UK in 1999, all new health technologies and clinical guidelines developed for the NHS are required to be assessed for cost-effectiveness. Quality-adjusted life years (QALYs), that combine both health related quality of life and life years into a single metric, is the standard outcome measure in economic evaluations. Generic health related quality of life is recommended to be measured using short self-complete questionnaires, with the EQ-5D measure recommended by NICE (NICE, 2014).

As NICE's remit continues to expand into broader areas such as public health and social care, there is increasing interest in looking at ways of incorporating additional information on patient benefit into cost-effectiveness analysis. There is increasing interest among health economists to measure outcomes from health and related interventions that assess broader wellbeing, allowing for cross-sectoral comparisons across health care and other public bodies (Brazier and Tsuchiya, 2015). One such approach in measuring broader wellbeing has focused on individual's capability to do and be the things in life that matter to them, as an alternative to focusing solely on

For peer review only - http://bmjopen.bmj.com/site/about/guidelines.xhtml

health status. Capability measures have been developed for the assessment of specific aspects of health and care, such as chronic pain (Kinghorn et al. 2015), public health (Lorgelly et al. 2015), mental health (Simon et al. 2013) and social care (Netten et al. 2012).

Another approach in measuring capability directly has been to develop short generic measures of perceived capability that could be applied across patient groups receiving health and social care interventions, but targeted to capture capability at different stages of life. The ICEpop CAPability measures, or ICECAP, attempt to capture the capability of all adults aged 18 years and older on the ICECAP-A (Al-Janabi et al. 2012), older adults who are aged 65 years and older on the ICECAP-O (Grewal et al. 2006, Coast et al. 2008) and more recently, a measure for those near the end of life, known as the supportive care measure or ICECAP-SCM (Sutton & Coast, 2014). Whereas the ICECAP-SCM has been designed specifically for programmes towards the end of life, the ICECAP-A and ICECAP-O allow for broader comparisons across health and social care interventions.

Both the ICECAP-A and ICECAP-O have five attributes of capability wellbeing (that is, wellbeing in terms of what people can "do" and "be") with three directly comparable attributes concerning "attachment", "enjoyment" and "autonomy (A)"/ "control (O)" and two less comparable attributes, albeit with overlapping themes: "achievement (A)" and "role (O)", and "stability (A)" and "security (O)" (see Table 1). Both instruments were developed using a similar qualitative interview process, where participants from the general public were asked to specify the aspects of quality of life that were of primary importance to them (Grewal et al. 2006, Al-Janabi et al. 2012). The descriptive system for both measures has four levels in each attribute, ranging from high to no capability, meaning 1024 (4^5) capability states are captured on both measures. Questions are phrased to capture a person's ability to achieve by asking whether an individual "can" or "is able" to achieve in different domains. Both the ICECAP-A and ICECAP-O are conceptually different from generic measures of health functioning, like the EQ-5D, commonly used in health economics (Davis et al. 2013, Keeley et al. 2016). The choice of self-complete questionnaire could also have important resource allocation implications, as recent

For peer review only - http://bmjopen.bmj.com/site/about/guidelines.xhtml

research suggests those with severe conditions and with depression are likely to receive greater priority when focusing on capability wellbeing (ICECAP-A) compared to health status (EQ-5D-5L) (Mitchell et al. 2015).

NICE have recently added the use of capability measures to their economic evaluation reference case concerning social care (NICE, 2014), and more recently, ICECAP measures have also been recommended for the economic assessment of interventions for long-term conditions in the Netherlands (Zorginstituut Nederland, 2015). Even though the ICECAP-A and ICECAP-O have overlapping themes, it is unclear as to what measure should be used to assess capability for patients requiring social care or living with a long-term health condition. Validity of both measures has taken place using qualitative and quantitative methods, but the comparative performance of both capability measures has not taken place.

Qualitative research validating the ICECAP-A has so far focused on members of the general population (Al-Janabi et al. 2013), and research professionals (Keeley et al. 2013). Some qualitative research has been conducted with the ICECAP-O in patient groups (Horwood et al. 2014, van Leeuwen et al. 2015). All four of these studies have used cognitive interview methods known as 'think aloud', whereby individuals are asked to verbalise their thought process when they are completing the measure (Willis, 2005). This process is thought to give a more realistic picture of the problems individuals face when completing questionnaires, than more probing interview methods that interrupt the task completion (Kuusela & Paul, 2000). Individuals are asked to verbalise their thought process as they complete the questionnaire, to examine the problems individuals encounter, in terms of comprehension, retrieval, judgement and response difficulties. The interviewer will remain silent throughout this process, so long as individuals continue to think out loud.

For peer review only - http://bmjopen.bmj.com/site/about/guidelines.xhtml

Table 1. Generic ICECAP measures: attributes and item descriptions

ICECAP-A (Al-Janabi et al. 2012)	ICECAP-O (Coast et al. 2008)
Stability	Attachment*
- an ability to feel settled and secure	- love and friendship
Attachment	Security
- an ability to have love, friendship and support	- thinking about the future without concern
Autonomy	Role
- an ability to be independent	- doing things that make you feel valued
Achievement	Enjoyment*
- an ability to achieve and progress in life	- enjoyment and pleasure
Enjoyment	Control
- an ability to experience enjoyment and pleasure	- an ability to be independent

*The autonomy (A) and control (O) attributes are phrased exactly the same on both measures. Attachment and enjoyment feature on both measures, but are phrased slightly differently. Stability (A) and security (O), and achievement (A) and role (O) are phrased differently in the descriptive system but have some overlapping themes in the qualitative analysis developing both measures.

The aim of this research is to explore the appropriateness of the ICECAP measures in people who require treatment for chronic kidney disease. In health economic analysis, there is interest in a common measure being used where appropriate, so that results across different patient populations have comparable outcomes when assessing cost-effectiveness. Although NICE has recently recommended the use of the ICECAP-O when assessing social care interventions (NICE, 2014), given that the ICECAP-A captures capability across a broader age range, an argument could be made for using the ICECAP-A for this reason. Patients with chronic kidney disease are likely to crossover the age range where both the ICECAP-A (18+) and ICECAP-O (65+) could feasibly be used. This study will therefore assess the appropriateness of each of the ICECAP measures in this patient population, based on the findings from the qualitative interviews.

For peer review only - http://bmjopen.bmj.com/site/about/guidelines.xhtml

It is also worth exploring how the completion of the ICECAP measures compare to the EQ-5D-5L, an expanded version (from three to five levels) of the recommended measure for generating QALYs by NICE. Each of these measures could be used in economic evaluations for patients with chronic kidney disease, so comparative information will be useful in this area. Qualitative interviews will allow for such comparisons, both based on the ease of completion of the measures by the patients in the think aloud exercise and also follow-up questioning on how each of the measures account for the patient's perspective of what aspects of quality of life are most important to them.

For peer review only

Study Objective

The objectives of this study are:

- To assess the feasibility of completing the ICECAP measures and the EQ-5D-5L for people receiving treatment for chronic kidney disease.
- To explore the difficulties in completing the three measures in terms of errors in terms of comprehending, retrieving, judging, responding and struggles (i.e. difficulty answering question, but eventually responded appropriately).
- To seek patient views as to how well the different questionnaires capture their quality of life.

Study Site

Patients will be recruited through the Richard Bright Renal Unit, Southmead Hospital Bristol.

Subjects and Recruitment

Inclusion criteria

To be included in the study, patients must meet *all* of the following criteria:

- Have chronic kidney disease (CKD stage 1-5)
- Willing and able to provide informed consent to participate
- Able to communicate in English

For peer review only - http://bmjopen.bmj.com/site/about/guidelines.xhtml

Exclusion criteria

To be excluded in the study, patients must meet *any* of the following criteria:

- Do not have chronic kidney disease (CKD stage 1-5)
- Is not willing and able to provide informed consent to participate
- Is not able to communicate in English

Ethical considerations and informed consent

Patients will be directly recruited through the Richard Bright Renal Unit, Southmead Hospital Bristol. A health care professional from the renal unit will identify patients meeting the inclusion criteria and determine whether the patient might wish to participate. Participants will be given the option of completing the interview in a private room at the renal unit or at their home at a time that is convenient for them. At the point of taking informed consent, the researcher (PM) will go through the information sheet with the participant, answer any questions and request informed consent, with this process expected to take approximately 10 minutes.

Sample size determination

Participants will be sampled using purposive sampling, with diversity sought in terms of age (<65 or >65), sex, and type of kidney care received. The study will aim to recruit approximately 25 patients to participate or until data saturation is reached, whereby no new themes are emerging from the interviews. There is no clear sample size for cognitive interviewing. Previous published think aloud studies using ICECAP measures have had sample sizes ranging from 10 (van Leeuwen et al. 2015) to 34 (Al-Janabi et al. 2013) participants. It is anticipated that a sample size of 25 should be adequate to enable the scoring of struggles and errors in the think aloud task, as well as identifying important themes from the interviews and conclusions about the use of the three quality of life measures for patients with chronic kidney disease.

Withdrawal of participants

Participants are free to withdraw from the study at any time. Clinical care *will not* be affected should the participant decide to withdraw from the study.

For peer review only - http://bmjopen.bmj.com/site/about/guidelines.xhtml

Study Design

Patients will be interviewed in a private room at the renal unit or in their home. The interview will commence with a recap of the study aims and an explanation of the format of the interview. Participants will be asked a number of questions concerning socio-demographic information such as age, sex, living alone, condition severity and if patients receive dialysis or not.

To get participants warmed-up, a simple think aloud task will be asked in relation to the number of windows an individual has in their house. Then the Global Quality of Life scale (Hyland & Sodergren 1996) will be presented to them as a practice of self-reporting and thinking out loud. Following the completion of the warm-up task, patients will be randomly allocated the ICECAP-A or the ICECAP-O first or last, with the EQ-5D-5L and EQ-VAS completed in between. Participants will not be interrupted unless they are silent for longer than 10 seconds when they will be asked to “keep thinking aloud”.

Following the completion of the three measures, a discussion between the researcher and participant will follow to clarify the informants’ thoughts whilst completing the measures. In particular, attention will be paid to difficulty in answering the different aspects of the measures and where there was judged to have been uncertainty in the response given by the participant.

The interview will conclude with a semi-structured interview format where patients will be asked about their views on the patient reported outcome measures they reported. Namely, interest will be given to measures they felt best captured their quality of life, what they liked about the measures and what aspects of their quality of life did they feel was missing from the questions being asked.

As the completion of self-reported measures of quality of life using the think aloud process can be emotional for the participants as they reflect on their quality of life, it can be a challenging experience for them. Participants will be offered breaks in the interview process if they are overwhelmed by emotions throughout the interview and will be given the option to stop the interview if that is what they would prefer.

For peer review only - http://bmjopen.bmj.com/site/about/guidelines.xhtml

Analysis Plan

All interviews will be transcribed verbatim and, from the transcript, three independent raters will code the transcripts with the aim of identifying four types of response problems to the measures, as well as any areas of 'struggle' (i.e. difficulty in answering that is not so severe as to constitute a response problem). Transcripts will be segmented to material relating to each of the attributes on the three measures. The four types of response problems that will be considered are: comprehension, retrieval, judgement and response. A standardised classification scheme will be employed to consistently identify four types of response problems. The classification is based on the survey response model, developed in cognitive psychology, that suggests that participants perform four actions when answering a question item with errors possible at each stage (Tourangeau et al. 2000). To appropriately answer a question using the survey response model, an individual must: (i) understand (comprehend) the question in the way that the researcher intended; (ii) successfully retrieve the appropriate information to answer the question from their long-term memory; (iii) correctly judge how the recalled information should be used to answer the question; and (iv) format the information into a valid response for the questionnaire.

Three raters (PM, FC and JC) will then independently code the 15 segments (5 items per measure) in each transcript as either: (a) error-free, (b) containing one or more errors or (c) as a 'struggle'. The struggle category is used to identify segments where the participant clearly has difficulty answering the question, but eventually reaches an appropriate answer. Consistency between raters on the coding of the data will be assessed using raw agreement and kappa statistics (Cohen, 1960). Following the independent coding, segments will be judged as errors (or struggles) if a majority of coders note a specific type of error (or struggle). Segments where two raters note a struggle or error but disagree on error type, will be discussed, with a code agreed upon by all raters.

Constant comparative methods will be used to derive explanatory themes from the interviews (Strauss & Corbin, 1990). Transcripts will be read and re-read, and categories and sub-categories will be developed to describe emerging themes. Descriptive accounts will be formed, and matrices used to aid comparison. Issues that are likely to be of interest include the *nature* of response problems across the

For peer review only - http://bmjopen.bmj.com/site/about/guidelines.xhtml

different measures, as well as the number of struggles and errors noted for the EQ-5D-5L compared to the ICECAP-A and the ICECAP-O. Themes will focus around reasoning behind preferred measures, aspects of measures they did not like and aspects of quality of life they felt were missing from all measures. Any other themes that arise during completion of the questionnaire and subsequent interview will also be examined.

Data Management

Confidentiality

Confidentiality of all information will be maintained in line with the Data Protection Act. Names and addresses of informants will not be linked to the data obtained and individuals will be identified on transcripts by means of a serial number only. Reporting of data will be in the form of anonymised quotes. Individuals will never be identified in person. Names and addresses of participants will not be released to any outside body or organisation.

Source documents

Interviews will be audio recorded and transcribed verbatim.

Records retention

Research data will include audio-tapes and computer files. Transcripts will be made of audio-tapes, at which point they will be anonymised and all identifiers and potential identifiers removed. Tapes will be kept in a locked filling cabinet and destroyed following the completion of transcribing and primary analysis of the interviews.

Sponsorship and ethical arrangements

Sponsorship of this research project is provided by the University of Bristol (study 2650). Ethics is sought from the NHS Research Ethics Committee.

For peer review only - http://bmjopen.bmj.com/site/about/guidelines.xhtml

Insurance

Liability insurance cover for this study is provided by the University of Bristol.

Publication Policy

This research will be written up for peer reviewed publication and submitted to a relevant journal, such as *Quality of Life Research, Social Science & Medicine* or *Value in Health* or a relevant renal journal interested in qualitative research and/or the measurement of quality of life. This study aims to provide valuable research information for a larger research fellowship proposal concerning the use of multiple outcomes in economic evaluations and how it can aid decision-making, with a case study developed in patients with end stage renal disease.

Study Personnel

Paul M. Mitchell, PhD, is a Senior Research Associate at the School of Social and Community Medicine, University of Bristol. Paul is currently funded through a postdoctoral research fellowship in health economics, jointly awarded by the UK Renal Registry and NIHR CLAHRC West.

Fergus J. Caskey, MBChB, MSc, MD, FRCP, is a Senior Clinical Lecturer at the School of Social and Community Medicine, University of Bristol, and Medical Director of the UK Renal Registry, Southmead Hospital Bristol. Dr Caskey is a consultant nephrologist who has vast experience of conducting research within the kidney patient population.

Joanna Coast, PhD, is a Professor of the Economics of Health and Care at the School of Social and Community Medicine, University of Bristol. Professor Coast has particular expertise in the application of qualitative methods, including think aloud studies, in health economics. She was the lead developer for the ICECAP capability measures.

Conflicts of interest

Joanna Coast was the lead developer for the ICECAP measures. Paul Mitchell and Fergus Caskey have no conflicts of interests to declare.

For peer review only - http://bmjopen.bmj.com/site/about/guidelines.xhtml

References

Al-Janabi, H., Flynn, T. N., & Coast, J. (2012). Development of a self-report measure of capability wellbeing for adults: the ICECAP-A. *Quality of Life Research*, 21, 167-176.
Al-Janabi, H., Keeley, T., Mitchell, P., & Coast, J. (2013). Can capabilities be self-reported? A think aloud study. *Social Science & Medicine*, 87, 116-122.
Brazier, J., & Tsuchiya, A. (2015). Improving Cross-Sector Comparisons: Going Beyond the Health-Related QALY. *Applied Health Economics and Health Policy*, 13, 557-565.
Coast, J., Flynn, T.N., Natarajan, L., Sproston, K., Lewis, J., Louviere, J.J., & Peters, T.J. (2008). Valuing the ICECAP capability index for older people. *Social Science & Medicine*, 67, 874-882.
Cohen, J. (1960). *A coefficient of agreement for nominal scales*. Educational and Psychological Measurement, 20, 37-46.
Davis, J.C., Liu-Ambrose, T., Richardson, C.G., & Byran S. (2013). A comparison of the ICECAP-O with EQ-5D in a falls prevention clinical setting: are they complements or substitutes? *Quality of Life Research*, 22, 969-977.
Grewal, I., Lewis, J., Flynn, T., Brown, J., Bond, J., & Coast, J. (2006). Developing attributes for a generic quality of life measure for older people: Preferences or capabilities? *Social Science & Medicine*, 62, 1891-1901.
Horwood, J., Sutton, E., & Coast, J. (2014). Evaluating the Face Validity of the ICECAP-O Capabilities Measure: A "Think Aloud" Study with Hip and Knee Arthoplasty Patients. *Applied Research in Quality of Life*, 9, 667-682.
Hyland, M.E., & Sodergren, S.C. (1996) Development of a new type of global quality of life scale, and comparison of performance and preference for 12 global scales. *Quality of Life Research*, 5, 469-480.
Keeley, T., Al-Janabi, H., Lorgelly, P., & Coast, J. (2013). A Qualitative Assessment of the Content Validity of the ICECAP-A and the EQ-5D-5L and Their Appropriateness for Use in Health
Keeley, T., Coast, J., Nicholls, E., Foster, N.E., Jowett, S., & Al-Janabi, H. (2016). An analysis of the complementarity of ICECAP-A and EQ-5D-3L in an adult population of patients with knee pain. *Health and Quality of Life Outcomes*, 14, 36.
Kinghorn, P., Robinson, A., & Smith, R.D. (2015). Developing a Capability-Based Questionnaire for Assessing Well-Being in Patients with Chronic Pain. *Social Indicators Research*, 120, 897-916.
Lorgelly, P., Lorimer, K., Fenwick, E.A.L., Briggs, A.H., & Anand, P. (2015). Operationalising the capability approach as an outcome measure in public health: The development of the OCAP-18. *Social Science & Medicine*, 142, 68-81.
Mitchell, P.M., Al-Janabi, H., Richardson, J., Iezzi, A., & Coast, J. (2015) The relative impacts of disease on health status and capability wellbeing: a multi-country study. *PLoS ONE*, 10, e0143590.
NICE (2014). Developing NICE guidelines: the manual. National Institute for Health and Care Excellence, London, UK, 123.
Netten, A., Burge, P., Malley, J., Potoglou, D., Towers, A-M., Brazier, J., Flynn, T., Forder, J., & Wall B. (2012). Outcomes of social care for adults: developing a preference-weighted measure. *Health Technology Assessment*, 16, 1-165.
Simon, J., Anand, P., Gray, A., Rugkåsa, J., Yeeles, K., & Burns T. (2013). Operationalising the capability approach for outcome measurement in mental health research. *Social Science & Medicine*, 98, 187-196.
Strauss, A., & Corbin, J. (1990). *Basics of qualitative research*. California, US: Sage Publications.

For peer review only - http://bmjopen.bmj.com/site/about/guidelines.xhtml

Sutton, E.J., & Coast, J. (2014). Development of a supportive care measure for economic evaluation of end-of-life care using qualitative methods. *Palliative Medicine*, 28, 151-157.
Tourangeau, R., Rips, L., & Rasinski, K. (2000). *The psychology of survey response*. New York: Cambridge University Press.
van Leeuwen, K.M., Jansen, A.P.D., Muntinga, M.E., Bosmans, J.E., Westerman, M.J., van Tulder, M.W., & van der Horst, H.E. (2015). Exploration of the content validity and feasibility of the EQ-5D-3L, ICECAP-O and ASCOT in older adults. *Value in Health*, 15, 201.
Willis, G. (2005). *Cognitive interviewing: A tool for improving questionnaire design*. Thousand Oaks, CA: Sage Publications.
Zorginstituut Nederland (2015). Richtlijn voor het uitvoeren van economische evaluaties in de gezondheidszorg. Web link: https://www.zorginstituutnederland.nl/binaries/content/documents/zinl-www/pakket/werkwijze-pakketbeheer/beoordeling-geneesmiddelen/farmaco-economisch-onderzoek/farmaco-economisch-onderzoek/farmaco-economisch-onderzoek/zinl%3Adocuments/1511-richtlijn-voor-het-uitvoeren-van-economische-evaluaties-in-de-gezondheidszorg/Richtlijn+voor+het+uitvoeren+van+economische+evaluaties+in+de+gezondheidszorg.pdf

For peer review only - http://bmjopen.bmj.com/site/about/guidelines.xhtml

Kidney patients' views on quality of life questionnaires

Participant Information Sheet

Information about the Research

We would like to invite you to take part in a research study that aims to understand how quality of life is measured in patient reported questionnaires. The study will be carried out by researchers from the University of Bristol together with the UK Renal Registry. To help you decide if you wish to take part, this leaflet explains the purpose of the research and how you would be involved. A member of our team will go through this information sheet with you and answer any questions you may have. This is likely to take about five to ten minutes.

What is the purpose of the study?

The aim of this study is to find out about what patients receiving kidney care think when answering quality of life questionnaires. Three quality of life measures are potentially useful for comparing the cost-effectiveness of different kidney care services. We are interested to know which measure you think best measures your quality of life.

Why have I been invited?

You have been invited because you are attending a clinic at the Richard Bright Renal Unit, Southmead Hospital Bristol. We hope that about 25 patients will take part.

Participation in the study is entirely up to you, but your help would be much appreciated. We will discuss the study with you and go through this information sheet before you make a decision. If you agree to take part, we will ask you to sign a consent form. One of our researchers will then arrange to interview you. You are free to withdraw from the study at any time and you do not need to tell us why. Please be assured that if you withdraw it will in no way affect the standard of care you receive.

For peer review only

What will I have to do?

First we will ask you a few questions about you and the type of kidney care you receive. We will then ask you to 'think aloud' as you complete three short questionnaires with a total of 15 questions. By 'thinking aloud' we mean we would like you to talk us through what you are thinking as you answer the questions. We will record the interview on tape to make sure your views are accurately reported. The interviewer will explain about 'thinking aloud' more fully at the start of the interview, and then have a chat with you at the end. We expect the interview to last no longer than 45 minutes. You are welcome to take breaks during the interview as and when you see fit.

What are the possible benefits of taking part?

Your views as a patient will provide important research input. You will help us understand if the questions asked in quality of life questionnaires are easy to understand and if the questions are important to your quality of life. You will also have a chance to discuss parts of quality of life that you do not think are being measured by these questionnaires.

Are there any disadvantages?

There is a small chance that you may find some of the research questions difficult to answer and upsetting to talk about your quality of life. You are free to withdraw from the interview at any time. We will destroy the interview recorded, if that is what you want.

Will my taking part in the study be kept confidential?

Yes. We will take great care to protect the confidentiality of the information you give us. If you agree to take part and have the interview recorded, we will use a non-personal code to identify the recording so that you cannot be recognised. Any names or places you mention during the interview will be anonymised. The questionnaire you complete will be identified by a study number. The recorded interview will be securely stored through the University of Bristol Research Data Storage Facility, at which point the tape recorded version on the pin protected digital recording device will be destroyed. Only members of the research team will have access to any information you provide during the interview.

For peer review only

For peer review only - http://bmjopen.bmj.com/site/about/guidelines.xhtml

What if there is a problem?

If you have any concern about any aspect of this study, please feel free to speak to one of the researchers (Dr. Paul Mitchell, Dr. Fergus Caskey, Dr. Jemima Scott or Professor Joanna Coast, who can be contacted through the School of Social and Community Medicine, University of Bristol, Canynge Hall, 39 Whatley Road, Bristol, BS8 2PS). If you wish to complain formally, you can send a written complaint to Patient advice and liaison services (PALS): Southmead Hospital and address it to Southmead Road, Westbury-on-Trym, Bristol, Avon, BS10 5NB.

What will happen to the results of the research study?

The results will be written up for researchers in medicine and the social sciences. The results will be useful in deciding what quality of life questionnaires to use when evaluating kidney care treatments. In deciding how to measure quality of life, we think it is important to ask people themselves what they think and not just rely on the views of professionals or the government.

Please be aware that all data we collect from this study will be retained for ten years, in accordance with standard University of Bristol data management practice. Anonymised quotations from this interview may also be used in study reports and publications.

Who is organising and funding the research?

This study is being carried out by the University of Bristol in conjunction with the Richard Bright Renal Unit, Southmead Hospital Bristol. The research team comprises three members. Dr Paul Mitchell is the lead researcher for this project. Dr Fergus Caskey is the Medical Director of the UK Renal Registry, a Consultant Nephrologist and a Senior Clinical Lecturer at the School of Social and Community Medicine, University of Bristol. Dr Jemima Scott is an Academic Clinical Fellow in renal care at Southmead Hospital Bristol. Professor Joanna Coast is a Professor in the Economics of Health and Care at the School of Social Science and Community Medicine, University of Bristol.

The research is funded through Paul's work with the UK Renal Registry who are based at Southmead Hospital Bristol and an organisation funded by the NHS to do health and care research called the NIHR CLAHRC West.

For peer review only - http://bmjopen.bmj.com/site/about/guidelines.xhtml

Who has reviewed the study?

All research in the NHS is looked at by an independent group of people, called a Research Ethics Committee, to protect your interests. This study has been reviewed and given a favourable opinion by the East of England – Cambridge Central Research Ethics Committee (REF: 16/EE/0331).

If you decide to participate you will have a copy of this information sheet and a signed consent form to keep.

Further information and contact details

Specific information about this research can be provided by lead researcher Dr. Paul Mitchell (e-mail: paul.mitchall@bristol.ac.uk or telephone number 0117 342 1264).

If you would like to receive a copy of the results of this study, please contact Paul using the contact details above. We expect the results of this study to be published by the end of 2018.

Following the interview you may find there are issues that have been discussed about which you would like further support. If this is the case, you can talk to staff in the renal unit where further services can be offered. Or you may find the following national resources helpful:

British Kidney Patient Association: www.britishkidney-pa.co.uk

Think Kidneys National Programme: www.thinkkidneys.nhs.uk

For peer review only

University of BRISTOL

RENAL ASSOCIATION UK RENAL REGISTRY

NHS National Institute for Health Research CLAHRC

For peer review only - http://bmjopen.bmj.com/site/about/guidelines.xhtml

COREQ (COnsolidated criteria for REporting Qualitative research) Checklist

A checklist of items that should be included in reports of qualitative research. You must report the page number in your manuscript where you consider each of the items listed in this checklist. If you have not included this information, either revise your manuscript accordingly before submitting or note N/A.

Topic	Item No.	Guide Questions/Description	Reported on Page No.
**Domain 1: Research team and reflexivity**			
Personal characteristics			
Interviewer/facilitator	1	Which author/s conducted the interview or focus group?	
Credentials	2	What were the researcher's credentials? E.g. PhD, MD	
Occupation	3	What was their occupation at the time of the study?	
Gender	4	Was the researcher male or female?	
Experience and training	5	What experience or training did the researcher have?	
Relationship with participants			
Relationship established	6	Was a relationship established prior to study commencement?	
Participant knowledge of the interviewer	7	What did the participants know about the researcher? e.g. personal goals, reasons for doing the research	
Interviewer characteristics	8	What characteristics were reported about the inter viewer/facilitator? e.g. Bias, assumptions, reasons and interests in the research topic	
**Domain 2: Study design**			
Theoretical framework			
Methodological orientation and Theory	9	What methodological orientation was stated to underpin the study? e.g. grounded theory, discourse analysis, ethnography, phenomenology, content analysis	
Participant selection			
Sampling	10	How were participants selected? e.g. purposive, convenience, consecutive, snowball	
Method of approach	11	How were participants approached? e.g. face-to-face, telephone, mail, email	
Sample size	12	How many participants were in the study?	
Non-participation	13	How many people refused to participate or dropped out? Reasons?	
Setting			
Setting of data collection	14	Where was the data collected? e.g. home, clinic, workplace	
Presence of non-participants	15	Was anyone else present besides the participants and researchers?	
Description of sample	16	What are the important characteristics of the sample? e.g. demographic data, date	
Data collection			
Interview guide	17	Were questions, prompts, guides provided by the authors? Was it pilot tested?	
Repeat interviews	18	Were repeat inter views carried out? If yes, how many?	
Audio/visual recording	19	Did the research use audio or visual recording to collect the data?	
Field notes	20	Were field notes made during and/or after the inter view or focus group?	
Duration	21	What was the duration of the inter views or focus group?	
Data saturation	22	Was data saturation discussed?	
Transcripts returned	23	Were transcripts returned to participants for comment and/or	

For peer review only

Topic	Item No.	Guide Questions/Description	Reported on Page No.
		correction?	
**Domain 3: analysis and findings**			
Data analysis			
Number of data coders	24	How many data coders coded the data?	
Description of the coding tree	25	Did authors provide a description of the coding tree?	
Derivation of themes	26	Were themes identified in advance or derived from the data?	
Software	27	What software, if applicable, was used to manage the data?	
Participant checking	28	Did participants provide feedback on the findings?	
Reporting			
Quotations presented	29	Were participant quotations presented to illustrate the themes/findings? Was each quotation identified? e.g. participant number	
Data and findings consistent	30	Was there consistency between the data presented and the findings?	
Clarity of major themes	31	Were major themes clearly presented in the findings?	
Clarity of minor themes	32	Is there a description of diverse cases or discussion of minor themes?	

Developed from: Tong A, Sainsbury P, Craig J. Consolidated criteria for reporting qualitative research (COREQ): a 32-item checklist for interviews and focus groups. *International Journal for Quality in Health Care*. 2007. Volume 19, Number 6: pp. 349 – 357

Once you have completed this checklist, please save a copy and upload it as part of your submission. DO NOT include this checklist as part of the main manuscript document. It must be uploaded as a separate file.

For peer review only

BMJ Open

Response process validity of three patient reported outcome measures for people requiring kidney care: a think-aloud study using the EQ-5D-5L, ICECAP-A and ICECAP-O

Journal:	*BMJ Open*
Manuscript ID	bmjopen-2019-034569.R1
Article Type:	Original research
Date Submitted by the Author:	10-Feb-2020
Complete List of Authors:	Mitchell , Paul; University of Bristol, Health Economics Bristol<br>Caskey, Fergus; University of Bristol, Population Health Sciences, University of Bristol; UK Renal Registry, Southmead Hospital Bristol<br>Scott, Jemima; UK Renal Registry, Southmead Hospital Bristol; Southmead Hospital, North Bristol NHS Trust<br>Sanghera, Sabina ; University of Bristol, Health Economics Bristol, Population Health Sciences<br>Coast, Joanna; University of Bristol, Health Economics Bristol, Population Health Sciences
**Primary Subject Heading**:	Health economics
Secondary Subject Heading:	Qualitative research
Keywords:	HEALTH ECONOMICS, capability approach, QUALITATIVE RESEARCH, quality of life, wellbeing, kidney care

SCHOLARONE™
Manuscripts

I, the Submitting Author has the right to grant and does grant on behalf of all authors of the Work (as defined in the below author licence), an exclusive licence and/or a non-exclusive licence for contributions from authors who are: i) UK Crown employees; ii) where BMJ has agreed a CC-BY licence shall apply, and/or iii) in accordance with the terms applicable for US Federal Government officers or employees acting as part of their official duties; on a worldwide, perpetual, irrevocable, royalty-free basis to BMJ Publishing Group Ltd ("BMJ") its licensees and where the relevant Journal is co-owned by BMJ to the co-owners of the Journal, to publish the Work in this journal and any other BMJ products and to exploit all rights, as set out in our licence.

The Submitting Author accepts and understands that any supply made under these terms is made by BMJ to the Submitting Author unless you are acting as an employee on behalf of your employer or a postgraduate student of an affiliated institution which is paying any applicable article publishing charge ("APC") for Open Access articles. Where the Submitting Author wishes to make the Work available on an Open Access basis (and intends to pay the relevant APC), the terms of reuse of such Open Access shall be governed by a Creative Commons licence – details of these licences and which Creative Commons licence will apply to this Work are set out in our licence referred to above.

Other than as permitted in any relevant BMJ Author's Self Archiving Policies, I confirm this Work has not been accepted for publication elsewhere, is not being considered for publication elsewhere and does not duplicate material already published. I confirm all authors consent to publication of this Work and authorise the granting of this licence.

For peer review only

Response process validity of three patient reported outcome measures for people requiring kidney care: a think-aloud study using the EQ-5D-5L, ICECAP-A and ICECAP-O

Mitchell, Paul Mark,[1*] Caskey, Fergus John,[2-4] Scott, Jemima,[3-4] Sanghera, Sabina,[1] Coast, Joanna[1]

Affiliations

1. Health Economics Bristol (HEB), Population Health Sciences, University of Bristol, Bristol, UK.
2. Population Health Sciences, University of Bristol, Bristol, UK
3. UK Renal Registry, Southmead Hospital Bristol, Bristol, UK.
4. Southmead Hospital Bristol, North Bristol NHS Trust, Bristol, UK

*** Corresponding author:**

E-mail: paul.mitchell@bristol.ac.uk

Phone: +44 (0)117 428 3122

Postal address: 1-5 Whiteladies Road (Room 2.05), Bristol, BS8 1NU, UK

Keywords: health economics; capability approach; qualitative research; quality of life; wellbeing

Word count – excluding title page, references, figures and tables: 4,235

For peer review only - http://bmjopen.bmj.com/site/about/guidelines.xhtml

Abstract

Objectives: To determine the response process validity, feasibility of completion, acceptability and preferences for three patient reported outcome measures that could be used in economic evaluation - the EQ-5D-5L, ICECAP-A and ICECAP-O in people requiring kidney care.

Design: Participants were asked to 'think-aloud' while completing the EQ-5D-5L, ICECAP-A and ICECAP-O, followed by a semi-structured interview. Five raters identified errors or struggles in completing the measures from the think-aloud component of the transcripts. Patient preferences for measures were extracted from the semi-structured interview.

Setting: Eligible patients were identified through a large UK secondary care renal centre.

Participants: In total, 30 participants were included in the study, consisting of patients attending renal outpatients for chronic kidney disease (n=18), with a functioning kidney transplant (n=6), and receiving haemodialysis (n=6).

Results: Participants had few errors and struggles in completing the EQ-5D-5L (11% error rate, 3% struggle rate), ICECAP-A (2% error rate, 2% struggle rate) and ICECAP-O (4% error rate, 3% struggle rate). The main errors with the EQ-5D-5L were judgements that did not comply with the "your health today" instruction. Comprehension errors were most prominent on ICECAP-O. Judgement errors were the only errors reported on ICECAP-A. Although the EQ-5D-5L had slightly more errors and struggles, it was the measure most preferred, with participants able to make a clearer link with EQ-5D-5L and their health condition.

Conclusions: The EQ-5D-5L, ICECAP-A and ICECAP-O are feasible for people requiring kidney care to complete and can be included in studies conducting economic evaluations of kidney care interventions. Further research is required to assess how health (e.g. EQ-5D) and capability (e.g. ICECAP) measures can be included in an economic evaluation simultaneously, as well as what ICECAP measure(s) to include when patient groups straddle the age ranges for ICECAP-A (18 years and older) and ICECAP-O (65 years and older).

Strengths and limitations of this study

- This is the first study to include EQ-5D-5L, ICECAP-A and ICECAP-O in a think-aloud study with people requiring kidney care
- The sample consists of a broad range of people requiring kidney care including renal outpatients for chronic kidney disease, kidney transplant check-ups and haemodialysis
- Think-aloud studies aim to identify errors and struggles in task completion as they occur
- Five raters, with diverse experience across health economics, qualitative research and kidney care, identified errors and struggles from the think-aloud transcripts
- Think-aloud relies on participants verbalising their difficulty in task completion

For peer review only - http://bmjopen.bmj.com/site/about/guidelines.xhtml

For peer review only

BACKGROUND

Healthcare expenditure is rising globally and has been increasing at a faster rate than international economic growth over the past decade.[1] Chronic kidney disease (CKD) is a growing burden on healthcare resources. In the 2015 Global Burden of Disease Study,[2] CKD were the twelfth leading cause of death and seventeenth leading cause of global life years lost.[3] In the UK alone, CKD accounts for more than one per cent of the national health service (NHS) annual budget.[4] Given this volume of expenditure, it is important that any healthcare resources allocated to managing kidney problems are used efficiently.

To determine which interventions should be recommended for practice, economic evaluations provide evidence on cost-effectiveness by comparing the costs and benefits of alternative interventions. In health and care, these economic evaluations increasingly rely on patient reported outcome measures (PROMs) to capture the health-related quality of life improvements from interventions[5] and are recommended for the generation of quality adjusted life years (QALYs) internationally.[6] A QALY is a combination of life years adjusted for health related quality of life.[7] Choice of PROM in generating QALYs plays an influential role in deciding if a treatment is cost-effective.[8 9] The EQ-5D is the most widely used measure to calculate QALYs in economic evaluations internationally [5] and has been translated into 169 different languages.[10] The EQ-5D has also been separately recommended by an expert consensus for routine collection across European renal registries.[11]

Despite international recommended use of QALYs in healthcare, the suitability of this outcome is debated, partly due to the exclusive focus on health gains and not broader wellbeing.[7 12 13] An alternative approach has been proposed to capture broader wellbeing, which focuses on a person's capabilities, meaning a person's freedom to achieve the things in life that are valuable to them.[14] Health bodies in the UK and the Netherlands have recognised the limitation of relying purely on QALYs in social care[15] and long-term health conditions.[16] Capability measures, such as the ICECAP-A[17] (A- all adults aged 18 years and above) and the ICECAP-O (O- older adults aged 65 years and above),[18] have been recommended as ways to capture the broader benefits for these patient groups. It is not entirely clear, however, which ICECAP measure to use when the age range of a patient group could use either ICECAP-A or ICECAP-O. People requiring kidney care are a prime example of this challenge, with the median age for starting renal replacement therapy in the UK being 64 years of age in 2017.[19] A recent study found the ICECAP-O to be a valid measure in over 75 year old patients receiving dialysis or conservative care for end stage kidney disease (ESKD).[20] ICECAP-O was also developed first and has been shown to be a valid outcome in older and younger adults in different settings.[21] However, no previous study has tested the ICECAP-A and ICECAP-O in the same patient group.[21]

For peer review only - http://bmjopen.bmj.com/site/about/guidelines.xhtml

The objective of this study was to (1) assess response process validity, feasibility of completion and acceptability of the health related quality of life PROM EQ-5D-5L,and two capability PROMs of broader wellbeing, ICECAP-A and ICECAP-O, in patients requiring kidney care, and (2) assess patient preferences for the three PROMs.

METHODS

This research consists of a ‘think-aloud’ study followed by a semi-structured interview. A think-aloud study is a cognitive interview method whereby individuals are asked to verbalise their thought process when completing measures.[22] Think-aloud interviews enable the examination of problems patients may encounter in terms of comprehension, retrieval, judgement and response difficulties. The interviewer remains silent, so long as individuals continue to think-aloud. This process is thought to give a more realistic picture of the problems that individuals face when completing questionnaires than more direct interview methods that interrupt task completion.[23] Think aloud interviews are a method that allow for the assessment of validity in terms of investigating response processes.[24] Assessing response processes are one of five recommended sources of validity evidence.[25] Ethics approval was obtained from the East of England NHS Research Ethics Committee (16/EE/0331) (see supplementary file 1 for research protocol).

Sampling and recruitment

Samples for previous think-aloud studies on health and capability PROMs have ranged from 10[26] to 34[27] participants. Based on these previous studies, saturation (whereby no new insights would be anticipated from additional sampling)[28] was expected to be reached at 25 participants here.

Patients were recruited through a large UK secondary care renal centre. Participants were sampled purposefully to achieve diversity in age (classified as <65 or ≥65) and type of kidney care received, but in line with general approaches to sampling in qualitative research, sampling did not aim for representativeness.[28] Sampling was conducted through renal outpatient lists and a dialysis unit. Eligibility required individuals to have chronic kidney disease, be willing and able to provide informed consent to participate, and be able to communicate in English (because the study was exploring the use of English language questionnaires). Potential participants received a participant information sheet (PIS – see supplementary file 2) in the post or at the dialysis unit and were invited to take part via a follow-up telephone call from a clinical trials officer. The PIS was the only information provided to the participant about the researcher prior to interview.

For peer review only - http://bmjopen.bmj.com/site/about/guidelines.xhtml

Instruments investigated

The EQ-5D-5L consists of five dimensions of health status covering mobility, self-care, usual activities, pain/discomfort and anxiety/depression across five levels ranging from no problems to extreme problems.[29] The EQ-5D-5L was introduced to supersede the EQ-5D-3L to reduce ceiling effects and increase sensitivity to change, by moving from a three level to a five level severity measure of health problems, but with the same five dimensions. A Visual Analogue Scale (VAS) is also included that asks respondents to rate their health today on a 0-100 scale from worst to best imaginable health state. The National Institute for Health and Care Excellence (NICE) in England recommends the collection of the EQ-5D-5L for conducting health economic evaluation.[30]

The ICECAP-A is a capability wellbeing measure developed for the general adult population (i.e. all adults, including those aged over 65).[17] It consists of five dimensions relating to a person's capability to have attachment, stability, achievement, enjoyment and autonomy. Each dimension has four levels ranging from no capability to full capability. The capabilities were identified through qualitative research with members of the general public aged 18 years and above (including over 65 year olds) to identify what was most important to them in their life.[17]

The ICECAP-O is a capability wellbeing measure developed for older adults.[18] The ICECAP-O was the first of the ICECAP suite of measures developed that aimed to develop a more appropriate quality of life measure for older adults specifically for use in the economic evaluation of health and care interventions.[31] It consists of five dimensions relating to a person's capability to have attachment, security, role, enjoyment and control. Each dimension has four levels ranging from no capability to full capability. As with the ICECAP-A, the capabilities were identified through qualitative research, but in this case with older members of the general public aged 65 years and above.[32]

Data collection

Once participants had provided informed consent, interviews took place at the renal centre or in the participant's home. All interviews were conducted by PM, a male PhD researcher in health economics with qualitative interview training and an interest in PROMs research. Initial questions focused on basic socio-demographic information. Participants then completed a simple warm-up task to determine the number of windows in their home. A second warm up task involved the completion of the Global Quality of Life scale.[33] Participants then completed the think-aloud exercise. They were allocated sequentially to receive ICECAP-A or ICECAP-O first or third, with EQ-5D-5L (including the EQ-VAS) always completed second; given the similarities between ICECAP-A and ICECAP-O, it was seen as a stronger design to separate these two measures to avoid confusion. Participants were not interrupted during the completion of the three measures unless they were silent for longer than 10

For peer review only - http://bmjopen.bmj.com/site/about/guidelines.xhtml

seconds when they were asked to "keep thinking aloud". Following the think-aloud task, a semi-structured interview was conducted to clarify issues arising in the think-aloud task and to explore views about the three measures. Field notes were made during the think-aloud component to guide the semi-structured interview. The interview guide was piloted prior to interview. Transcripts were not returned to participants for comment and/or correction and they did not provide feedback on the study findings. Repeat interviews were not carried out. Interviews were audio recorded and transcribed verbatim. Data were managed in Microsoft Word and Excel.

Data analysis

i. Think-aloud analysis

The think-aloud section from each transcript was extracted for each of the measures and divided into 16 segments: 6 representing the items on the EQ-5D-5L (including the EQ-5D-VAS), 5 items on the ICECAP-A and ICECAP-O, respectively. Think-aloud sections of the interview, alongside the reported response level for each item on each measure, were presented to five independent raters (PM, FC, JS, SS and JC), with expertise in health economics (PM, SS and JC), qualitative research (JC) and renal care (FC and JS). Each rater individually examined all think-aloud sections to identify problems participants encountered when completing each of the three measures. Raters were asked to identify whether responses were error free or contained any of the following problems, based on the survey response model [34]:

1. Comprehension error (understanding the question in the way the researcher intended)
2. Retrieval error (retrieving appropriate information from their long-term memory)
3. Judgement error (correctly judging how recalled information should be used to answer)
4. Response error (format the information into a valid response for the questionnaire)
5. Struggle (not one of the four errors but clear difficulty in answering the question) [27]

Following these independent ratings, each item was identified as error free, containing an error or containing a struggle, using the following rules:

- Where three or more raters identified a specific error/struggle it was classed as an error/struggle;
- Where one or none thought an error was present it was marked as error free;
- Where two or more raters identified an error/struggle but there was no majority agreement on the type of error/struggle, a decision was made during a consensus meeting with all raters; a majority decision was used when no consensus occurred.

For peer review only - http://bmjopen.bmj.com/site/about/guidelines.xhtml

Consistency between raters on the coding of the data was assessed using raw agreement and a weighted kappa statistic.[35] For the latter, where an error and no error were reported between raters, this was weighted as 0; all other disagreements - such as different error types, error/struggle or struggle/no error - was weighted as 0.5; with agreement weighted as 1.

ii. Preference between measures

During the semi-structured interviews following the think-aloud task, individual preferences for completing the three measures were explored. Individuals were asked which of the three measures they preferred and why they thought it was more important in assessing their quality of life.

Patient and public involvement

Patients and the public were not directly involved in the design of the study.

RESULTS

334 patients were invited to take part in the study. Of these, 161 responded to telephone follow-up and 37 agreed to participate. In four cases, patients did not attend the interview, one individual was too unwell to participate and one decided against participation during the consent process. Thirty-one individuals took part, but one individual did not understand the task (reading aloud their response levels only), leaving 30 individuals as the final sample. Most interviews took place at the health care facility, with four taking place in the participant's home. Most interviews were conducted one-to-one; on occasion at the health care facility patients' partners were present. Interviews were conducted between April and July 2017 and lasted between 16 and 55 minutes (average 33 minutes). Characteristics of the sample are presented in Table 1.

Think-aloud analysis: Errors and struggles

Following independent coding of the think-aloud interviews by five raters, inter-rater agreement was similar for ICECAP-A (85%-95%) and ICECAP-O (83%-93%), slightly lower for EQ-5D-5L (78%-84%) and weighted chance-corrected agreement being rated 'fair' to 'moderate' for 29 out of 30 inter-rater comparisons using standard guidelines.[36] Eight errors (four EQ-5D-5L, zero ICECAP-A and four ICECAP-O), eight struggles (five EQ-5D-5L, one ICECAP-A and two ICECAP-O) and 52 possible error/struggles were identified through independent rating. At the subsequent rater meeting, from the 52 possible error/struggles, a further 26 errors or struggles were agreed upon: 17 of 29 for the EQ-5D-5L, five of 11 for the ICECAP-A and four of 12 for the ICECAP-O. Breakdowns of error type by measure item are reported in tables 2-4.

For peer review only - http://bmjopen.bmj.com/site/about/guidelines.xhtml

Table 1 Participants' characteristics (n=30)

Sex	
Male	23
Female	7
Ethnicity	
White	28
Non-white	2
Age group	
75+	4
65-74	8
55-64	7
45-54	6
35-44	4
18-34	1
Kidney care received	
Renal outpatients	18
Renal outpatients (transplant)	6
Dialysis	6

Table 2 Errors and Struggles: EQ-5D-5L (n=30)

	Mobility	Self-Care	Usual Activities	Pain/ Discomfort	Anxiety/ Depression	VAS*	Total
Error							
Comprehension	0	0	1	0	0	0	1
Retrieval	0	0	0	0	0	0	0
Judgement	1	1	2	6	2	2	14
Response	0	0	2	2	0	1	5
Struggle	1	0	1	2	0	2	6
Total	2	1	6	10	2	5	26

*n=29

Table 3. Errors and Struggles: ICECAP-A (n=30)

	Stability	Attachment	Autonomy	Achievement	Enjoyment	Total
Error						
Comprehension	0	0	0	0	0	0
Retrieval	0	0	0	0	0	0
Judgement	0	0	2	1	0	3
Response	0	0	0	0	0	0
Struggle	2	0	0	1	0	3
Total	2	0	2	2	0	6

Table 4. Errors and Struggles: ICECAP-O (n=30)

	Attachment	Security	Role	Enjoyment	Control	Total
Error						
Comprehension	1	0	2	0	0	3
Retrieval	0	0	0	0	0	0
Judgement	0	0	1	0	0	1
Response	0	0	0	2	0	2
Struggle	1	1	2	0	0	4
Total	2	1	5	2	0	10

For peer review only

For peer review only - http://bmjopen.bmj.com/site/about/guidelines.xhtml

In total, 179 segments were generated for the EQ-5D-5L (one VAS was not completed by accident) and 150 segments each for the ICECAP-A and ICECAP-O. Twenty (11%) out of the 179 segments of the EQ-5D-5L were associated with an error and six (3%) with a struggle. Three (2%) out of the 150 segments of the ICECAP-A were associated with an error and three (2%) with a struggle. Six (4%) out of the 150 segments of the ICECAP-O were associated with an error and four (3%) with a struggle.

The majority of responses were not identified as an error or struggle on any of the three measures, indicating feasibility of use for all three PROMs. Participants found all measures easy to complete overall, showing acceptability in completing these PROMs:

Very straightforward. (Participant 26, male, aged 65-74, dialysis patient)

Not particularly, they all seemed, they're all pretty relevant to the questionnaire and to my condition and recovery and all that sort of thing so nothing sort of surprised me what was being asked so, happy with all the questions that was fine.

(Participant 21, male, 18-35, kidney transplant outpatient)

There were more errors (17) and struggles (4) reported for EQ-5D-5L (even when excluding the EQ-VAS) than for either ICECAP measure. The most common error type for EQ-5D-5L related to judgment, with this error recorded at least once across all EQ-5D-5L dimensions. Raters decided that a judgement error had occurred when participants clearly diverged from the EQ-5D-5L instruction to focus on "***your health today***":

I am working – I am doing this on – on a bad day.

(Participant 19, male, aged 55-64, judgement errors for four of five EQ-5D-5L dimensions)

Response errors for the pain/discomfort dimension were driven by the infrequency with which they were reported to occur:

But sitting here now I would put my state, a little bit of discomfort, but I don't think either end of the spectrum really indicates what I actually feel. Because it is a thing which either comes on and then is put right by antibiotics or painkillers, so I'm going to put moderate pain or discomfort. But perhaps there should be a box for occasional to indicate recurrent or occasional pain. (Participant 12, male, aged 65-74, pain/discomfort response error)

For peer review only - http://bmjopen.bmj.com/site/about/guidelines.xhtml

Response errors for usual activities were due to no response being provided and one participant felt that their true response was in between slight and moderate problems. The only other error recorded on the EQ-5D-5L was also for usual activities in terms of comprehension:

> *Not sure what my usual activities are. Walking I suppose. I'm sorry I can't think what my usual activities are. So I don't know what to put there.*
>
> (Participant 27, male, aged 75+, usual activities response error)

For the ICECAP-A, there were only judgement errors or struggles reported. The attachment and enjoyment dimensions were error and struggle free. Two of the judgement errors follow a similar pattern as for EQ-5D, where one individual reported their capability on a bad day, rather than at the moment. The other judgement error related to the participants' interpretation of the item:

> *… I'm reading that one as being completely independent, is that I would probably be quite happy living on my own rather than with a partner or family…*
>
> (Participant 10, male, aged 65-74, judgement error)

Comprehension errors were the highest error type for ICECAP-O, with two participants unable to understand the role dimension and one participant the attachment attribute:

> *Question one, love and friendship, reading the supposed answers, I find them rather confusing. I can have all of the love and friendship that I want (-) not really [sure] what the question is asking. Very difficult. Totally bemused by question one, so I will hazard a guess.*
>
> (Participant 8, male, aged 55-64, attachment comprehension error)
>
> *I don't want to feel valued. Again I don't understand what this means really. Valued by whom? (-) I don't know I can't answer that at all.*
>
> (Participant 27, male, aged 75+, role comprehension error)

Another error on the role attribute was found when one participant focused on functioning (i.e. what they do) rather than their capability (i.e. what they are able to do):

> *Yeah, actually, it's interesting if I think about it a bit more actually. I probably am able to do all of the things that make me feel valued but don't actually do them. I think I'll leave that to many of the things.* (Participant 10, male, aged 65-74, role judgement error)

For peer review only - http://bmjopen.bmj.com/site/about/guidelines.xhtml

There were two response errors on the enjoyment attribute where both individuals felt they were in between the same two levels:

> *…don't have all the time that I'd like to spend doing stuff outside the work so my answer's probably a two and a half but I'll put a three.*
>
> (Participant 16, male, aged 55-64, enjoyment response error)

> *Enjoyment and pleasure? I think I'm somewhere – I'm gonna put myself unhelpfully at two and a half because I don't think I have a little, I don't think I have a lot.*
>
> (Participant 18, male, aged 35-44, enjoyment response error)

Measure Preferences

The EQ-5D-5L was most preferred (n=17), five preferred ICECAP-O, three preferred ICECAP-A and five were unable to make a choice. One reason for preferring EQ-5D-5L was that participants could more clearly see the connection between the questions being asked and their illness:

> *I think the one that…because I've come via the kidney clinic, I'm-I'm thinking that this kidney research rather than general life research, so I think the one that relates most clearly to health and different problems that you might experience with kidney problems… is (EQ-5D-5L).*
>
> (Participant 5, female, aged 45-54)

> *That one about the physical thing. That seemed to be more relevant about whether you're well, ill or what other problems you've got. More relevant for a medical questionnaire rather than how you feel and stuff. But I know how you feel is important as well but you know, whether you can get about and might need help getting to appointments, things like that might be more, more relevant.* (Participant 17, male, aged 35-44)

For peer review only - http://bmjopen.bmj.com/site/about/guidelines.xhtml

For peer review only

Reasons for preferring either ICECAP measure were due to what was being measured and a perceived greater depth compared to the EQ-5D-5L:

> *That one's (EQ-5D-5L) really quite a superficial, can I walk around, can I wash myself, kind of very operational stuff. These two are more about kind of more psychological as well as quite physical things. Other than you talk about anxiety, depression there and I instantly said I don't – clearly they're in my head I'm not depressed, I don't have that illness. I would – so would put down to these two and I would go with this one (ICECAP-A) because I quite like the – the independent, achievement and progress but I think that one was a brilliant question because I think that's probably the most important thing that is on my mind at the moment.*
>
> (Participant 18, male, aged 35-44)

> *Because (ICECAP-O) that's… it's all embodying isn't it about your family, your life, what you do, where you think you're going.* (Participant 15, male, aged 65-74)

DISCUSSION

This study explored the response process validity, feasibility of completion and acceptability of EQ-5D-5L, ICECAP-A and ICECAP-O in patients requiring kidney care and preferences between the three PROMs. There were more errors and struggles reported with the EQ-5D-5L, mainly related to judgement errors with respect to the answer provided varying from the measure recall period "*your health today*". Nevertheless, most participants preferred the EQ-5D-5L for reasons of ease of completion and were more directly able to link the wording of the questions to their health condition. ICECAP-A had the fewest errors and struggles overall. One in six participants or more recorded an error or struggle in completing EQ-5D-5L pain/discomfort, EQ-5D-5L usual activities, EQ-5D-5L VAS and ICECAP-O role items.

For peer review only - http://bmjopen.bmj.com/site/about/guidelines.xhtml

This study is the first to collect both ICECAP-A and ICECAP-O measures simultaneously from the same population. The study benefits from having participants with a broad range of kidney problems and receiving different treatments. The heterogeneity of the sample in terms of age and treatment type means that the findings in this study could be applied to other similar settings. There are some limitations, however: the sample was predominantly male and of white ethnicity. Although most respondents were male, this is not dissimilar to renal replacement therapy recipients in the UK where almost two in every three patients (64.1%) are male.[19] Nevertheless, the findings need to be interpreted in light of the sample. In addition, the sample does not include patients receiving peritoneal dialysis or conservative care for ESKD. The think-aloud interview method also relies on participants verbalising their difficulty in task completion, so difficulties in completion that the participants did not or were not able to express are not captured here.

As with other similar size studies in different populations, this work has shown that responses to the ICECAP-A measure have fewer errors or struggles than those to EQ-5D. [27 37] It differs from the only existing comparison between EQ-5D-5L and ICECAP-O which, in a smaller study (n=10) found the EQ-5D-5L produced fewer errors in completion.[26] Errors associated with comprehending the attachment and role items on ICECAP-O are similar to previous think-aloud studies.[26 38] A potential concern over the use of the new EQ-5D-5L is the number of judgement errors that were found here. This seems to be particularly related to the prevalence of intermittent health problems for people requiring kidney care, which caused patients difficulty in responding particularly for the pain/discomfort dimension.

The findings suggest that all three measures are appropriate for use in people requiring kidney care, with low errors and struggles across all measures reflecting the feasibility and acceptability of the three PROMs in this sample. However, the three PROMs have different strengths and weaknesses; the fewer errors reported for the two ICECAP measures may be traded against the patients' preferences for the EQ-5D-5L. Indeed, the finding that the EQ-5D-5L was preferred by patients reflects earlier works showing that patients preferred EQ-5D over a number of other condition-specific and generic measures of health status.[11] For ICECAP measures, the ICECAP-A produced fewest errors across this population requiring kidney care covering a wide range of ages, but a recent study specifically aimed at over 75 year olds requiring treatment for ESKD found ICECAP-O to be a valid outcome.[20]

For peer review only - http://bmjopen.bmj.com/site/about/guidelines.xhtml

From a health and care decision-making point of view, although both errors in completion and patient preferences are important in choice of measure, they are unlikely to be the only considerations for choice of measure to aid in resource allocation decisions across health and care service provision. In a recent review of EQ-5D scores (i.e. using population preferences to value the relative importance of health states[7]) attached to health states for calculating QALYs in patients with ESKD, there is only a clear benefit attached to the health gain from kidney transplantation compared to other treatments, such as dialysis and conservative care.[39] This finding may not be surprising given health levels for people with kidney transplants have found to be comparable to that of the general population[40] and is generally considered the clinical "gold standard" treatment option for people with ESKD.[41] However, what may be surprising is that the EQ-5D is not able to distinguish patient benefits from the type of dialysis, how dialysis is delivered or whether dialysis is delivered at all. Previous stated preference research from Australia has shown that pre-dialysis patients would be willing to trade-off on average seven months of survival time to reduce the number of trips to hospital for dialysis per week and on average 15 months of survival time to reduce their restrictions on their ability to travel and make short trips.[42] Such important considerations do not appear to be captured using the current economic toolkit that focus primarily on patient health status and not the impact of that treatment on their broader ability to do and be things in life that matter to them.

Future work could look at how decision-makers can use health and capability measures simultaneously in an economic evaluation. In particular for kidney care, areas where capabilities might differ most from health measures like EQ-5D could be in areas where dialysis is delivered outside of a health care facility (i.e. peritoneal dialysis or home-based haemodialysis) or not delivered at all (i.e. conservative care). This study also highlights issues surrounding the variation in interpretation and judgements relating to the framing of EQ-5D (i.e. "*your health today*") and is likely to be of interest to explore further.[43]

Further research is required to better understand whether the different ICECAP measures are completed differently depending on the respondents' stage of life. Measuring capability at different stages across the life course may provide an alternative framework for using the ICECAP capability measures in economic evaluations for health and care interventions.[44] More detailed qualitative analysis of think-aloud and semi-structured interviews may provide some answers in the implementation of such a life course framework.

For peer review only - http://bmjopen.bmj.com/site/about/guidelines.xhtml

Contributorship statement

PM, FC and JC developed the study design. PM, FC and JS were involved in data acquisition. All authors were involved with analysing and interpreting the data. PM initially drafted this paper. All authors (PM, FC, JS, SS, JC) were involved in the revision of the initial draft for important intellectual content and final approval of this version to be published. All authors agree to be accountable for all aspects of the work in ensuring that questions related to the accuracy or integrity of any part of the work are appropriately investigated and resolved.

Competing interests

Joanna Coast led the development of the ICECAP-O and ICECAP-A. All other authors have no competing interests to declare.

Funding

This work was initially supported by a post-doctoral fellowship co-funded by the National Institute for Health Research (NIHR) Collaboration for Leadership in Applied Health Research and Care West (NIHR CLAHRC West) and the UK Renal Registry (UKRR). The views expressed in this article are those of the authors and not necessarily those of the NHS, the NIHR, the Department of Health and Social Care or the UKRR. Time for drafting this paper for Paul Mitchell and Joanna Coast has also been supported through a Wellcome Trust investigator award (205384/Z/16/Z).

Data sharing statement

No data are available.

Acknowledgements

We would like to thank Louise Hawkins for her help in participant recruitment and all the participants who took part in this think-aloud study. We would also like to thank Paula Lorgelly and Rachael Morton for providing an expert peer review of study protocol, Amanda Owen-Smith for taking part in a pilot think-aloud interview and the two peer reviewers for this paper. Finally, we thank Hugh McLeod for leading a discussion at the UK Health Economists' Study Group (HESG) in Bristol in June 2018 and feedback received from delegates at HESG and European Health Economics Association (EuHEA) Conference in Maastricht, the Netherlands, in July 2018.

For peer review only - http://bmjopen.bmj.com/site/about/guidelines.xhtml

For peer review only

REFERENCES

1. Xu K, Soucat A, Kutzin J, et al. Public Spending on Health: A Closer Look at Global Trends. Switzerland: World Health Organisation 2018.
2. Wang H, Naghavi M, Allen C, et al. Global, regional, and national life expectancy, all-cause mortality, and cause-specific mortality for 249 causes of death, 1980–2015: a systematic analysis for the Global Burden of Disease Study 2015. *The Lancet* 2016;388(10053):1459-544. doi: https://doi.org/10.1016/S0140-6736(16)31012-1
3. Neuen BL, Chadban SJ, Demaio AR, et al. Chronic kidney disease and the global NCDs agenda. 2017;2(2):e000380. doi: 10.1136/bmjgh-2017-000380 %J BMJ Global Health
4. Kerr M, Bray B, Medcalf J, et al. Estimating the financial cost of chronic kidney disease to the NHS in England. *Nephrology Dialysis Transplantation* 2012;27(suppl 3):iii73-iii80. doi: 10.1093/ndt/gfs269
5. Wisløff T, Hagen G, Hamidi V, et al. Estimating QALY Gains in Applied Studies: A Review of Cost-Utility Analyses Published in 2010. *Pharmacoeconomics* 2014;32(4):367-75. doi: 10.1007/s40273-014-0136-z
6. Rowen D, Azzabi Zouraq I, Chevrou-Severac H, et al. International Regulations and Recommendations for Utility Data for Health Technology Assessment. *PharmacoEconomics* 2017;35(1):11-19. doi: 10.1007/s40273-017-0544-y
7. Weinstein MC, Torrance G, McGuire A. QALYs: The Basics. *Value in Health* 2009;12:S5-S9. doi: 10.1111/j.1524-4733.2009.00515.x
8. Richardson J, Khan MA, Iezzi A, et al. Comparing and Explaining Differences in the Magnitude, Content, and Sensitivity of Utilities Predicted by the EQ-5D, SF-6D, HUI 3, 15D, QWB, and AQoL-8D Multiattribute Utility Instruments. *Medical Decision Making* 2014;35(3):276-91. doi: 10.1177/0272989X14543107
9. Hernandez Alava M, Wailoo A, Grimm S, et al. EQ-5D-5L versus EQ-5D-3L: The Impact on Cost Effectiveness in the United Kingdom. *Value in Health* 2018;21(1):49-56. doi: https://doi.org/10.1016/j.jval.2017.09.004
10. Devlin NJ, Krabbe PFM. The development of new research methods for the valuation of EQ-5D-5L. *The European Journal of Health Economics* 2013;14(1):1-3. doi: 10.1007/s10198-013-0502-3
11. Breckenridge K, Bekker HL, Gibbons E, et al. How to routinely collect data on patient-reported outcome and experience measures in renal registries in Europe: an expert consensus meeting. *Nephrology Dialysis Transplantation* 2015;30(10):1605-14. doi: 10.1093/ndt/gfv209
12. Coast J, Smith RD, Lorgelly P. Should the capability approach be applied in Health Economics? *Health Economics* 2008;17(6):667-70. doi: 10.1002/hec.1359
13. Dolan P, Kahneman D. Interpretations Of Utility And Their Implications For The Valuation Of Health*. *The Economic Journal* 2008;118(525):215-34. doi: 10.1111/j.1468-0297.2007.02110.x
14. Sen A. Capability and Well-Being. In: Nussbaum M, Sen A, eds. The Quality of Life. Oxford: Oxford University Press 1993:30-53.
15. NICE. NICE guidelines: the manual. London: National Institute for Health and Care Excellence, 2018:125.
16. ZorginstituutNederland. Guideline for economic evaluations in healthcare, 2016.
17. Al-Janabi H, Flynn T, Coast J. Development of a self-report measure of capability wellbeing for adults: the ICECAP-A. *Quality of Life Research* 2012;21(1):167-76. doi: 10.1007/s11136-011-9927-2
18. Coast J, Flynn TN, Natarajan L, et al. Valuing the ICECAP capability index for older people. *Social Science & Medicine* 2008;67(5):874-82. doi: https://doi.org/10.1016/j.socscimed.2008.05.015
19. UKRenalRegistry. UK Renal Registry 21st Annual Report - data to 31/12/2017. Bristol, UK: UK Renal Registry, 2019.

For peer review only - http://bmjopen.bmj.com/site/about/guidelines.xhtml

20. Shah KK, Murtagh FEM, McGeechan K, et al. Health-related quality of life and well-being in people over 75 years of age with end-stage kidney disease managed with dialysis or comprehensive conservative care: a cross-sectional study in the UK and Australia. *BMJ Open* 2019;9(5):e027776. doi: 10.1136/bmjopen-2018-027776
21. Proud L, McLoughlin C, Kinghorn P. ICECAP-O, the current state of play: a systematic review of studies reporting the psychometric properties and use of the instrument over the decade since its publication. *Quality of Life Research* 2019;28(6):1429-39. doi: 10.1007/s11136-019-02114-y
22. Willis GB. Cognitive interviewing: A tool for improving questionnaire design: Sage Publications 2004.
23. Kuusela H, Paul P. A Comparison of Concurrent and Retrospective Verbal Protocol Analysis. *The American Journal of Psychology* 2000;113(3):387-404. doi: 10.2307/1423365
24. Zumbo BD, Hubley AM. Understanding and investigating response processes in validation research: Springer 2017.
25. AERA, APA, NCME. Standards for educational and psychological testing: American Educational Research Association Washington, DC, 2014.
26. van Leeuwen KM, Jansen APD, Muntinga ME, et al. Exploration of the content validity and feasibility of the EQ-5D-3L, ICECAP-O and ASCOT in older adults. *BMC Health Services Research* 2015;15(1):201. doi: 10.1186/s12913-015-0862-8
27. Al-Janabi H, Keeley T, Mitchell P, et al. Can capabilities be self-reported? A think aloud study. *Social Science & Medicine* 2013;87:116-22. doi: https://doi.org/10.1016/j.socscimed.2013.03.035
28. Owen-Smith A, Coast J. Understanding sampling and recruitment. In: Coast J, ed. Qualitative Methods in Health Economics. London, UK: Rowman & Littlefield Ltd 2017:42-58.
29. Herdman M, Gudex C, Lloyd A, et al. Development and preliminary testing of the new five-level version of EQ-5D (EQ-5D-5L). *Quality of Life Research* 2011;20(10):1727-36. doi: 10.1007/s11136-011-9903-x
30. NICE. Position statement on the EQ-5D-5L value set for England online: National Institute for Health and Care Excellence; 2019 [updated October 2019. Available from: https://www.nice.org.uk/about/what-we-do/our-programmes/nice-guidance/technology-appraisal-guidance/eq-5d-5l accessed 24/01/2020.
31. Coast J, Kinghorn P, Mitchell P. The Development of Capability Measures in Health Economics: Opportunities, Challenges and Progress. *The Patient - Patient-Centered Outcomes Research* 2015;8(2):119-26. doi: 10.1007/s40271-014-0080-1
32. Grewal I, Lewis J, Flynn T, et al. Developing attributes for a generic quality of life measure for older people: Preferences or capabilities? *Social Science & Medicine* 2006;62(8):1891-901. doi: https://doi.org/10.1016/j.socscimed.2005.08.023
33. Hyland ME, Sodergren SC. Development of a new type of global quality of life scale, and comparison of performance and preference for 12 global scales. *Quality of Life Research* 1996;5(5):469-80. doi: 10.1007/bf00540019
34. Tourangeau R, Rips LJ, Rasinski K. The psychology of survey response: Cambridge University Press 2000.
35. Cohen J. A Coefficient of Agreement for Nominal Scales. *Educational and Psychological Measurement* 1960;20(1):37-46. doi: 10.1177/001316446002000104
36. Altman DG. Practical statistics for medical research. London: Chapman & Hall 1991.
37. Bailey C, Kinghorn P, Orlando R, et al. 'The ICECAP-SCM tells you more about what I'm going through': A think-aloud study measuring quality of life among patients receiving supportive and palliative care. *Palliative Medicine* 2016;30(7):642-52. doi: 10.1177/0269216315624890
38. Horwood J, Sutton E, Coast J. Evaluating the Face Validity of the ICECAP-O Capabilities Measure: A "Think Aloud" Study with Hip and Knee Arthroplasty Patients. *Applied Research in Quality of Life* 2014;9(3):667-82. doi: 10.1007/s11482-013-9264-4

For peer review only - http://bmjopen.bmj.com/site/about/guidelines.xhtml

39. Wyld M, Morton RL, Hayen A, et al. A Systematic Review and Meta-Analysis of Utility-Based Quality of Life in Chronic Kidney Disease Treatments. *PLoS Medicine* 2012;9(9):e1001307. doi: 10.1371/journal.pmed.1001307
40. Villeneuve C, Laroche M-L, Essig M, et al. Evolution and Determinants of Health-Related Quality-of-Life in Kidney Transplant Patients Over the First 3 Years After Transplantation. *Transplantation* 2016;100(3):640-47. doi: 10.1097/tp.0000000000000846
41. Wolfe RA, Ashby VB, Milford EL, et al. Comparison of Mortality in All Patients on Dialysis, Patients on Dialysis Awaiting Transplantation, and Recipients of a First Cadaveric Transplant. *New England Journal of Medicine* 1999;341(23):1725-30. doi: doi:10.1056/NEJM199912023412303
42. Morton RL, Snelling P, Webster AC, et al. Factors influencing patient choice of dialysis versus conservative care to treat end-stage kidney disease. *Canadian Medical Association Journal* 2012;184(5):E277-E83. doi: 10.1503/cmaj.111355
43. Sanghera S, Coast J. Measuring Quality-Adjusted Life-Years When Health Fluctuates. *Value in Health* 2019 doi: https://doi.org/10.1016/j.jval.2019.09.2753
44. Coast J. Assessing capability in economic evaluation: a life course approach? *The European Journal of Health Economics* 2019 doi: 10.1007/s10198-018-1027-6

For peer review only

For peer review only - http://bmjopen.bmj.com/site/about/guidelines.xhtml

School of Social and Community Medicine
NIHR CLAHRC West, 9th Floor Whitefriars
BRISTOL BS1 2NT
United Kingdom

+44.117.3421264
paul.mitchell@bristol.ac.uk
www.bristol.ac.uk/social-community-medicine/people/paul-m-mitchell/index.html

For peer review only

STUDY PROTOCOL

Kidney patients' views on quality of life questionnaires: a 'think-aloud' study

Paul M. Mitchell, Fergus J. Caskey, Joanna Coast

Background Information

Since the establishment of the National Institute for Health and Care Excellence (NICE) in the UK in 1999, all new health technologies and clinical guidelines developed for the NHS are required to be assessed for cost-effectiveness. Quality-adjusted life years (QALYs), that combine both health related quality of life and life years into a single metric, is the standard outcome measure in economic evaluations. Generic health related quality of life is recommended to be measured using short self-complete questionnaires, with the EQ-5D measure recommended by NICE (NICE, 2014).

As NICE's remit continues to expand into broader areas such as public health and social care, there is increasing interest in looking at ways of incorporating additional information on patient benefit into cost-effectiveness analysis. There is increasing interest among health economists to measure outcomes from health and related interventions that assess broader wellbeing, allowing for cross-sectoral comparisons across health care and other public bodies (Brazier and Tsuchiya, 2015). One such approach in measuring broader wellbeing has focused on individual's capability to do and be the things in life that matter to them, as an alternative to focusing solely on

For peer review only - http://bmjopen.bmj.com/site/about/guidelines.xhtml

health status. Capability measures have been developed for the assessment of specific aspects of health and care, such as chronic pain (Kinghorn et al. 2015), public health (Lorgelly et al. 2015), mental health (Simon et al. 2013) and social care (Netten et al. 2012).

Another approach in measuring capability directly has been to develop short generic measures of perceived capability that could be applied across patient groups receiving health and social care interventions, but targeted to capture capability at different stages of life. The ICEpop CAPability measures, or ICECAP, attempt to capture the capability of all adults aged 18 years and older on the ICECAP-A (Al-Janabi et al. 2012), older adults who are aged 65 years and older on the ICECAP-O (Grewal et al. 2006, Coast et al. 2008) and more recently, a measure for those near the end of life, known as the supportive care measure or ICECAP-SCM (Sutton & Coast, 2014). Whereas the ICECAP-SCM has been designed specifically for programmes towards the end of life, the ICECAP-A and ICECAP-O allow for broader comparisons across health and social care interventions.

Both the ICECAP-A and ICECAP-O have five attributes of capability wellbeing (that is, wellbeing in terms of what people can "do" and "be") with three directly comparable attributes concerning "attachment", "enjoyment" and "autonomy (A)"/ "control (O)" and two less comparable attributes, albeit with overlapping themes: "achievement (A)" and "role (O)", and "stability (A)" and "security (O)" (see Table 1). Both instruments were developed using a similar qualitative interview process, where participants from the general public were asked to specify the aspects of quality of life that were of primary importance to them (Grewal et al. 2006, Al-Janabi et al. 2012). The descriptive system for both measures has four levels in each attribute, ranging from high to no capability, meaning 1024 (4^5) capability states are captured on both measures. Questions are phrased to capture a person's ability to achieve by asking whether an individual "can" or "is able" to achieve in different domains. Both the ICECAP-A and ICECAP-O are conceptually different from generic measures of health functioning, like the EQ-5D, commonly used in health economics (Davis et al. 2013, Keeley et al. 2016). The choice of self-complete questionnaire could also have important resource allocation implications, as recent

For peer review only - http://bmjopen.bmj.com/site/about/guidelines.xhtml

research suggests those with severe conditions and with depression are likely to receive greater priority when focusing on capability wellbeing (ICECAP-A) compared to health status (EQ-5D-5L) (Mitchell et al. 2015).

NICE have recently added the use of capability measures to their economic evaluation reference case concerning social care (NICE, 2014), and more recently, ICECAP measures have also been recommended for the economic assessment of interventions for long-term conditions in the Netherlands (Zorginstituut Nederland, 2015). Even though the ICECAP-A and ICECAP-O have overlapping themes, it is unclear as to what measure should be used to assess capability for patients requiring social care or living with a long-term health condition. Validity of both measures has taken place using qualitative and quantitative methods, but the comparative performance of both capability measures has not taken place.

Qualitative research validating the ICECAP-A has so far focused on members of the general population (Al-Janabi et al. 2013), and research professionals (Keeley et al. 2013). Some qualitative research has been conducted with the ICECAP-O in patient groups (Horwood et al. 2014, van Leeuwen et al. 2015). All four of these studies have used cognitive interview methods known as ‘think aloud’, whereby individuals are asked to verbalise their thought process when they are completing the measure (Willis, 2005). This process is thought to give a more realistic picture of the problems individuals face when completing questionnaires, than more probing interview methods that interrupt the task completion (Kuusela & Paul, 2000). Individuals are asked to verbalise their thought process as they complete the questionnaire, to examine the problems individuals encounter, in terms of comprehension, retrieval, judgement and response difficulties. The interviewer will remain silent throughout this process, so long as individuals continue to think out loud.

For peer review only - http://bmjopen.bmj.com/site/about/guidelines.xhtml

Table 1. Generic ICECAP measures: attributes and item descriptions

ICECAP-A (Al-Janabi et al. 2012)	ICECAP-O (Coast et al. 2008)
Stability	Attachment*
- an ability to feel settled and secure	- love and friendship
Attachment	Security
- an ability to have love, friendship and support	- thinking about the future without concern
Autonomy	Role
- an ability to be independent	- doing things that make you feel valued
Achievement	Enjoyment*
- an ability to achieve and progress in life	- enjoyment and pleasure
Enjoyment	Control
- an ability to experience enjoyment and pleasure	- an ability to be independent

*The autonomy (A) and control (O) attributes are phrased exactly the same on both measures. Attachment and enjoyment feature on both measures, but are phrased slightly differently. Stability (A) and security (O), and achievement (A) and role (O) are phrased differently in the descriptive system but have some overlapping themes in the qualitative analysis developing both measures.

The aim of this research is to explore the appropriateness of the ICECAP measures in people who require treatment for chronic kidney disease. In health economic analysis, there is interest in a common measure being used where appropriate, so that results across different patient populations have comparable outcomes when assessing cost-effectiveness. Although NICE has recently recommended the use of the ICECAP-O when assessing social care interventions (NICE, 2014), given that the ICECAP-A captures capability across a broader age range, an argument could be made for using the ICECAP-A for this reason. Patients with chronic kidney disease are likely to crossover the age range where both the ICECAP-A (18+) and ICECAP-O (65+) could feasibly be used. This study will therefore assess the appropriateness of each of the ICECAP measures in this patient population, based on the findings from the qualitative interviews.

For peer review only - http://bmjopen.bmj.com/site/about/guidelines.xhtml

It is also worth exploring how the completion of the ICECAP measures compare to the EQ-5D-5L, an expanded version (from three to five levels) of the recommended measure for generating QALYs by NICE. Each of these measures could be used in economic evaluations for patients with chronic kidney disease, so comparative information will be useful in this area. Qualitative interviews will allow for such comparisons, both based on the ease of completion of the measures by the patients in the think aloud exercise and also follow-up questioning on how each of the measures account for the patient's perspective of what aspects of quality of life are most important to them.

Study Objective

The objectives of this study are:

- To assess the feasibility of completing the ICECAP measures and the EQ-5D-5L for people receiving treatment for chronic kidney disease.
- To explore the difficulties in completing the three measures in terms of errors in terms of comprehending, retrieving, judging, responding and struggles (i.e. difficulty answering question, but eventually responded appropriately).
- To seek patient views as to how well the different questionnaires capture their quality of life.

Study Site

Patients will be recruited through the Richard Bright Renal Unit, Southmead Hospital Bristol.

Subjects and Recruitment

Inclusion criteria

To be included in the study, patients must meet *all* of the following criteria:

- Have chronic kidney disease (CKD stage 1-5)
- Willing and able to provide informed consent to participate
- Able to communicate in English

For peer review only - http://bmjopen.bmj.com/site/about/guidelines.xhtml

Exclusion criteria

To be excluded in the study, patients must meet *any* of the following criteria:

- Do not have chronic kidney disease (CKD stage 1-5)
- Is not willing and able to provide informed consent to participate
- Is not able to communicate in English

Ethical considerations and informed consent

Patients will be directly recruited through the Richard Bright Renal Unit, Southmead Hospital Bristol. A health care professional from the renal unit will identify patients meeting the inclusion criteria and determine whether the patient might wish to participate. Participants will be given the option of completing the interview in a private room at the renal unit or at their home at a time that is convenient for them. At the point of taking informed consent, the researcher (PM) will go through the information sheet with the participant, answer any questions and request informed consent, with this process expected to take approximately 10 minutes.

Sample size determination

Participants will be sampled using purposive sampling, with diversity sought in terms of age (<65 or >65), sex, and type of kidney care received. The study will aim to recruit approximately 25 patients to participate or until data saturation is reached, whereby no new themes are emerging from the interviews. There is no clear sample size for cognitive interviewing. Previous published think aloud studies using ICECAP measures have had sample sizes ranging from 10 (van Leeuwen et al. 2015) to 34 (Al-Janabi et al. 2013) participants. It is anticipated that a sample size of 25 should be adequate to enable the scoring of struggles and errors in the think aloud task, as well as identifying important themes from the interviews and conclusions about the use of the three quality of life measures for patients with chronic kidney disease.

Withdrawal of participants

Participants are free to withdraw from the study at any time. Clinical care *will not* be affected should the participant decide to withdraw from the study.

For peer review only - http://bmjopen.bmj.com/site/about/guidelines.xhtml

Study Design

Patients will be interviewed in a private room at the renal unit or in their home. The interview will commence with a recap of the study aims and an explanation of the format of the interview. Participants will be asked a number of questions concerning socio-demographic information such as age, sex, living alone, condition severity and if patients receive dialysis or not.

To get participants warmed-up, a simple think aloud task will be asked in relation to the number of windows an individual has in their house. Then the Global Quality of Life scale (Hyland & Sodergren 1996) will be presented to them as a practice of self-reporting and thinking out loud. Following the completion of the warm-up task, patients will be randomly allocated the ICECAP-A or the ICECAP-O first or last, with the EQ-5D-5L and EQ-VAS completed in between. Participants will not be interrupted unless they are silent for longer than 10 seconds when they will be asked to "keep thinking aloud".

Following the completion of the three measures, a discussion between the researcher and participant will follow to clarify the informants' thoughts whilst completing the measures. In particular, attention will be paid to difficulty in answering the different aspects of the measures and where there was judged to have been uncertainty in the response given by the participant.

The interview will conclude with a semi-structured interview format where patients will be asked about their views on the patient reported outcome measures they reported. Namely, interest will be given to measures they felt best captured their quality of life, what they liked about the measures and what aspects of their quality of life did they feel was missing from the questions being asked.

As the completion of self-reported measures of quality of life using the think aloud process can be emotional for the participants as they reflect on their quality of life, it can be a challenging experience for them. Participants will be offered breaks in the interview process if they are overwhelmed by emotions throughout the interview and will be given the option to stop the interview if that is what they would prefer.

For peer review only - http://bmjopen.bmj.com/site/about/guidelines.xhtml

Analysis Plan

All interviews will be transcribed verbatim and, from the transcript, three independent raters will code the transcripts with the aim of identifying four types of response problems to the measures, as well as any areas of 'struggle' (i.e. difficulty in answering that is not so severe as to constitute a response problem). Transcripts will be segmented to material relating to each of the attributes on the three measures. The four types of response problems that will be considered are: comprehension, retrieval, judgement and response. A standardised classification scheme will be employed to consistently identify four types of response problems. The classification is based on the survey response model, developed in cognitive psychology, that suggests that participants perform four actions when answering a question item with errors possible at each stage (Tourangeau et al. 2000). To appropriately answer a question using the survey response model, an individual must: (i) understand (comprehend) the question in the way that the researcher intended; (ii) successfully retrieve the appropriate information to answer the question from their long-term memory; (iii) correctly judge how the recalled information should be used to answer the question; and (iv) format the information into a valid response for the questionnaire.

Three raters (PM, FC and JC) will then independently code the 15 segments (5 items per measure) in each transcript as either: (a) error-free, (b) containing one or more errors or (c) as a 'struggle'. The struggle category is used to identify segments where the participant clearly has difficulty answering the question, but eventually reaches an appropriate answer. Consistency between raters on the coding of the data will be assessed using raw agreement and kappa statistics (Cohen, 1960). Following the independent coding, segments will be judged as errors (or struggles) if a majority of coders note a specific type of error (or struggle). Segments where two raters note a struggle or error but disagree on error type, will be discussed, with a code agreed upon by all raters.

Constant comparative methods will be used to derive explanatory themes from the interviews (Strauss & Corbin, 1990). Transcripts will be read and re-read, and categories and sub-categories will be developed to describe emerging themes. Descriptive accounts will be formed, and matrices used to aid comparison. Issues that are likely to be of interest include the *nature* of response problems across the

For peer review only - http://bmjopen.bmj.com/site/about/guidelines.xhtml

different measures, as well as the number of struggles and errors noted for the EQ-5D-5L compared to the ICECAP-A and the ICECAP-O. Themes will focus around reasoning behind preferred measures, aspects of measures they did not like and aspects of quality of life they felt were missing from all measures. Any other themes that arise during completion of the questionnaire and subsequent interview will also be examined.

Data Management

Confidentiality

Confidentiality of all information will be maintained in line with the Data Protection Act. Names and addresses of informants will not be linked to the data obtained and individuals will be identified on transcripts by means of a serial number only. Reporting of data will be in the form of anonymised quotes. Individuals will never be identified in person. Names and addresses of participants will not be released to any outside body or organisation.

Source documents

Interviews will be audio recorded and transcribed verbatim.

Records retention

Research data will include audio-tapes and computer files. Transcripts will be made of audio-tapes, at which point they will be anonymised and all identifiers and potential identifiers removed. Tapes will be kept in a locked filling cabinet and destroyed following the completion of transcribing and primary analysis of the interviews.

Sponsorship and ethical arrangements

Sponsorship of this research project is provided by the University of Bristol (study 2650). Ethics is sought from the NHS Research Ethics Committee.

For peer review only - http://bmjopen.bmj.com/site/about/guidelines.xhtml

Insurance

Liability insurance cover for this study is provided by the University of Bristol.

Publication Policy

This research will be written up for peer reviewed publication and submitted to a relevant journal, such as *Quality of Life Research*, *Social Science & Medicine* or *Value in Health* or a relevant renal journal interested in qualitative research and/or the measurement of quality of life. This study aims to provide valuable research information for a larger research fellowship proposal concerning the use of multiple outcomes in economic evaluations and how it can aid decision-making, with a case study developed in patients with end stage renal disease.

Study Personnel

Paul M. Mitchell, PhD, is a Senior Research Associate at the School of Social and Community Medicine, University of Bristol. Paul is currently funded through a postdoctoral research fellowship in health economics, jointly awarded by the UK Renal Registry and NIHR CLAHRC West.

Fergus J. Caskey, MBChB, MSc, MD, FRCP, is a Senior Clinical Lecturer at the School of Social and Community Medicine, University of Bristol, and Medical Director of the UK Renal Registry, Southmead Hospital Bristol. Dr Caskey is a consultant nephrologist who has vast experience of conducting research within the kidney patient population.

Joanna Coast, PhD, is a Professor of the Economics of Health and Care at the School of Social and Community Medicine, University of Bristol. Professor Coast has particular expertise in the application of qualitative methods, including think aloud studies, in health economics. She was the lead developer for the ICECAP capability measures.

Conflicts of interest

Joanna Coast was the lead developer for the ICECAP measures. Paul Mitchell and Fergus Caskey have no conflicts of interests to declare.

For peer review only - http://bmjopen.bmj.com/site/about/guidelines.xhtml

References

Al-Janabi, H., Flynn, T. N., & Coast, J. (2012). Development of a self-report measure of capability wellbeing for adults: the ICECAP-A. *Quality of Life Research*, 21, 167-176.

Al-Janabi, H., Keeley, T., Mitchell, P., & Coast, J. (2013). Can capabilities be self-reported? A think aloud study. *Social Science & Medicine*, 87, 116-122.

Brazier, J., & Tsuchiya, A. (2015). Improving Cross-Sector Comparisons: Going Beyond the Health-Related QALY. *Applied Health Economics and Health Policy*, 13, 557-565.

Coast, J., Flynn, T.N., Natarajan, L., Sproston, K., Lewis, J., Louviere, J.J., & Peters, T.J. (2008). Valuing the ICECAP capability index for older people. *Social Science & Medicine*, 67, 874-882.

Cohen, J. (1960). *A coefficient of agreement for nominal scales*. Educational and Psychological Measurement, 20, 37-46.

Davis, J.C., Liu-Ambrose, T., Richardson, C.G., & Byran S. (2013). A comparison of the ICECAP-O with EQ-5D in a falls prevention clinical setting: are they complements or substitutes? *Quality of Life Research*, 22, 969-977.

Grewal, I., Lewis, J., Flynn, T., Brown, J., Bond, J., & Coast, J. (2006). Developing attributes for a generic quality of life measure for older people: Preferences or capabilities? *Social Science & Medicine*, 62, 1891-1901.

Horwood, J., Sutton, E., & Coast, J. (2014). Evaluating the Face Validity of the ICECAP-O Capabilities Measure: A "Think Aloud" Study with Hip and Knee Arthoplasty Patients. *Applied Research in Quality of Life*, 9, 667-682.

Hyland, M.E., & Sodergren, S.C. (1996) Development of a new type of global quality of life scale, and comparison of performance and preference for 12 global scales. *Quality of Life Research*, 5, 469-480.

Keeley, T., Al-Janabi, H., Lorgelly, P., & Coast, J. (2013). A Qualitative Assessment of the Content Validity of the ICECAP-A and the EQ-5D-5L and Their Appropriateness for Use in Health

Keeley, T., Coast, J., Nicholls, E., Foster, N.E., Jowett, S., & Al-Janabi, H. (2016). An analysis of the complementarity of ICECAP-A and EQ-5D-3L in an adult population of patients with knee pain. *Health and Quality of Life Outcomes*, 14, 36.

Kinghorn, P., Robinson, A., & Smith, R.D. (2015). Developing a Capability-Based Questionnaire for Assessing Well-Being in Patients with Chronic Pain. *Social Indicators Research*, 120, 897-916.

Lorgelly, P., Lorimer, K., Fenwick, E.A.L., Briggs, A.H., & Anand, P. (2015). Operationalising the capability approach as an outcome measure in public health: The development of the OCAP-18. *Social Science & Medicine*, 142, 68-81.

Mitchell, P.M., Al-Janabi, H., Richardson, J., Iezzi, A., & Coast, J. (2015) The relative impacts of disease on health status and capability wellbeing: a multi-country study. *PLoS ONE*, 10, e0143590.

NICE (2014). Developing NICE guidelines: the manual. National Institute for Health and Care Excellence, London, UK, 123.

Netten, A., Burge, P., Malley, J., Potoglou, D., Towers, A-M., Brazier, J., Flynn, T., Forder, J., & Wall B. (2012). Outcomes of social care for adults: developing a preference-weighted measure. *Health Technology Assessment*, 16, 1-165.

Simon, J., Anand, P., Gray, A., Rugkåsa, J., Yeeles, K., & Burns T. (2013). Operationalising the capability approach for outcome measurement in mental health research. *Social Science & Medicine*, 98, 187-196.

Strauss, A., & Corbin, J. (1990). *Basics of qualitative research*. California, US: Sage Publications.

For peer review only - http://bmjopen.bmj.com/site/about/guidelines.xhtml

Sutton, E.J., & Coast, J. (2014). Development of a supportive care measure for economic evaluation of end-of-life care using qualitative methods. *Palliative Medicine*, 28, 151-157.
Tourangeau, R., Rips, L., & Rasinski, K. (2000). *The psychology of survey response*. New York: Cambridge University Press.
van Leeuwen, K.M., Jansen, A.P.D., Muntinga, M.E., Bosmans, J.E., Westerman, M.J., van Tulder, M.W., & van der Horst, H.E. (2015). Exploration of the content validity and feasibility of the EQ-5D-3L, ICECAP-O and ASCOT in older adults. *Value in Health*, 15, 201.
Willis, G. (2005). *Cognitive interviewing: A tool for improving questionnaire design*. Thousand Oaks, CA: Sage Publications.
Zorginstituut Nederland (2015). Richtlijn voor het uitvoeren van economische evaluaties in de gezondheidszorg. Web link: https://www.zorginstituutnederland.nl/binaries/content/documents/zinl-www/pakket/werkwijze-pakketbeheer/beoordeling-geneesmiddelen/farmaco-economisch-onderzoek/farmaco-economisch-onderzoek/farmaco-economisch-onderzoek/zinl%3Adocuments/1511-richtlijn-voor-het-uitvoeren-van-economische-evaluaties-in-de-gezondheidszorg/Richtlijn+voor+het+uitvoeren+van+economische+evaluaties+in+de+gezondheidszorg.pdf

For peer review only - http://bmjopen.bmj.com/site/about/guidelines.xhtml

Kidney patients' views on quality of life questionnaires

Participant Information Sheet

Information about the Research

We would like to invite you to take part in a research study that aims to understand how quality of life is measured in patient reported questionnaires. The study will be carried out by researchers from the University of Bristol together with the UK Renal Registry. To help you decide if you wish to take part, this leaflet explains the purpose of the research and how you would be involved. A member of our team will go through this information sheet with you and answer any questions you may have. This is likely to take about five to ten minutes.

What is the purpose of the study?

The aim of this study is to find out about what patients receiving kidney care think when answering quality of life questionnaires. Three quality of life measures are potentially useful for comparing the cost-effectiveness of different kidney care services. We are interested to know which measure you think best measures your quality of life.

Why have I been invited?

You have been invited because you are attending a clinic at the Richard Bright Renal Unit, Southmead Hospital Bristol. We hope that about 25 patients will take part.

Participation in the study is entirely up to you, but your help would be much appreciated. We will discuss the study with you and go through this information sheet before you make a decision. If you agree to take part, we will ask you to sign a consent form. One of our researchers will then arrange to interview you. You are free to withdraw from the study at any time and you do not need to tell us why. Please be assured that if you withdraw it will in no way affect the standard of care you receive.

For peer review only

For peer review only - http://bmjopen.bmj.com/site/about/guidelines.xhtml

What will I have to do?

First we will ask you a few questions about you and the type of kidney care you receive. We will then ask you to 'think aloud' as you complete three short questionnaires with a total of 15 questions. By 'thinking aloud' we mean we would like you to talk us through what you are thinking as you answer the questions. We will record the interview on tape to make sure your views are accurately reported. The interviewer will explain about 'thinking aloud' more fully at the start of the interview, and then have a chat with you at the end. We expect the interview to last no longer than 45 minutes. You are welcome to take breaks during the interview as and when you see fit.

What are the possible benefits of taking part?

Your views as a patient will provide important research input. You will help us understand if the questions asked in quality of life questionnaires are easy to understand and if the questions are important to your quality of life. You will also have a chance to discuss parts of quality of life that you do not think are being measured by these questionnaires.

Are there any disadvantages?

There is a small chance that you may find some of the research questions difficult to answer and upsetting to talk about your quality of life. You are free to withdraw from the interview at any time. We will destroy the interview recorded, if that is what you want.

Will my taking part in the study be kept confidential?

Yes. We will take great care to protect the confidentiality of the information you give us. If you agree to take part and have the interview recorded, we will use a non-personal code to identify the recording so that you cannot be recognised. Any names or places you mention during the interview will be anonymised. The questionnaire you complete will be identified by a study number. The recorded interview will be securely stored through the University of Bristol Research Data Storage Facility, at which point the tape recorded version on the pin protected digital recording device will be destroyed. Only members of the research team will have access to any information you provide during the interview.

For peer review only

For peer review only - http://bmjopen.bmj.com/site/about/guidelines.xhtml

What if there is a problem?

If you have any concern about any aspect of this study, please feel free to speak to one of the researchers (Dr. Paul Mitchell, Dr. Fergus Caskey, Dr. Jemima Scott or Professor Joanna Coast, who can be contacted through the School of Social and Community Medicine, University of Bristol, Canynge Hall, 39 Whatley Road, Bristol, BS8 2PS). If you wish to complain formally, you can send a written complaint to Patient advice and liaison services (PALS): Southmead Hospital and address it to Southmead Road, Westbury-on-Trym, Bristol, Avon, BS10 5NB.

What will happen to the results of the research study?

The results will be written up for researchers in medicine and the social sciences. The results will be useful in deciding what quality of life questionnaires to use when evaluating kidney care treatments. In deciding how to measure quality of life, we think it is important to ask people themselves what they think and not just rely on the views of professionals or the government.

Please be aware that all data we collect from this study will be retained for ten years, in accordance with standard University of Bristol data management practice. Anonymised quotations from this interview may also be used in study reports and publications.

Who is organising and funding the research?

This study is being carried out by the University of Bristol in conjunction with the Richard Bright Renal Unit, Southmead Hospital Bristol. The research team comprises three members. Dr Paul Mitchell is the lead researcher for this project. Dr Fergus Caskey is the Medical Director of the UK Renal Registry, a Consultant Nephrologist and a Senior Clinical Lecturer at the School of Social and Community Medicine, University of Bristol. Dr Jemima Scott is an Academic Clinical Fellow in renal care at Southmead Hospital Bristol. Professor Joanna Coast is a Professor in the Economics of Health and Care at the School of Social Science and Community Medicine, University of Bristol.

The research is funded through Paul's work with the UK Renal Registry who are based at Southmead Hospital Bristol and an organisation funded by the NHS to do health and care research called the NIHR CLAHRC West.

For peer review only

For peer review only - http://bmjopen.bmj.com/site/about/guidelines.xhtml

Who has reviewed the study?

All research in the NHS is looked at by an independent group of people, called a Research Ethics Committee, to protect your interests. This study has been reviewed and given a favourable opinion by the East of England – Cambridge Central Research Ethics Committee (REF: 16/EE/0331).

If you decide to participate you will have a copy of this information sheet and a signed consent form to keep.

Further information and contact details

Specific information about this research can be provided by lead researcher Dr. Paul Mitchell (e-mail: paul.mitchall@bristol.ac.uk or telephone number 0117 342 1264).

If you would like to receive a copy of the results of this study, please contact Paul using the contact details above. We expect the results of this study to be published by the end of 2018.

Following the interview you may find there are issues that have been discussed about which you would like further support. If this is the case, you can talk to staff in the renal unit where further services can be offered. Or you may find the following national resources helpful:

British Kidney Patient Association: www.britishkidney-pa.co.uk

Think Kidneys National Programme: www.thinkkidneys.nhs.uk

For peer review only

For peer review only - http://bmjopen.bmj.com/site/about/guidelines.xhtml

COREQ (COnsolidated criteria for REporting Qualitative research) Checklist

A checklist of items that should be included in reports of qualitative research. You must report the page number in your manuscript where you consider each of the items listed in this checklist. If you have not included this information, either revise your manuscript accordingly before submitting or note N/A.

Topic	Item No.	Guide Questions/Description	Reported on Page No.
**Domain 1: Research team and reflexivity**			
Personal characteristics			
Interviewer/facilitator	1	Which author/s conducted the interview or focus group?	
Credentials	2	What were the researcher's credentials? E.g. PhD, MD	
Occupation	3	What was their occupation at the time of the study?	
Gender	4	Was the researcher male or female?	
Experience and training	5	What experience or training did the researcher have?	
Relationship with participants			
Relationship established	6	Was a relationship established prior to study commencement?	
Participant knowledge of the interviewer	7	What did the participants know about the researcher? e.g. personal goals, reasons for doing the research	
Interviewer characteristics	8	What characteristics were reported about the inter viewer/facilitator? e.g. Bias, assumptions, reasons and interests in the research topic	
**Domain 2: Study design**			
Theoretical framework			
Methodological orientation and Theory	9	What methodological orientation was stated to underpin the study? e.g. grounded theory, discourse analysis, ethnography, phenomenology, content analysis	
Participant selection			
Sampling	10	How were participants selected? e.g. purposive, convenience, consecutive, snowball	
Method of approach	11	How were participants approached? e.g. face-to-face, telephone, mail, email	
Sample size	12	How many participants were in the study?	
Non-participation	13	How many people refused to participate or dropped out? Reasons?	
Setting			
Setting of data collection	14	Where was the data collected? e.g. home, clinic, workplace	
Presence of non-participants	15	Was anyone else present besides the participants and researchers?	
Description of sample	16	What are the important characteristics of the sample? e.g. demographic data, date	
Data collection			
Interview guide	17	Were questions, prompts, guides provided by the authors? Was it pilot tested?	
Repeat interviews	18	Were repeat inter views carried out? If yes, how many?	
Audio/visual recording	19	Did the research use audio or visual recording to collect the data?	
Field notes	20	Were field notes made during and/or after the inter view or focus group?	
Duration	21	What was the duration of the inter views or focus group?	
Data saturation	22	Was data saturation discussed?	
Transcripts returned	23	Were transcripts returned to participants for comment and/or	

For peer review only

Topic	Item No.	Guide Questions/Description	Reported on Page No.
		correction?	
**Domain 3: analysis and findings**			
Data analysis			
Number of data coders	24	How many data coders coded the data?	
Description of the coding tree	25	Did authors provide a description of the coding tree?	
Derivation of themes	26	Were themes identified in advance or derived from the data?	
Software	27	What software, if applicable, was used to manage the data?	
Participant checking	28	Did participants provide feedback on the findings?	
Reporting			
Quotations presented	29	Were participant quotations presented to illustrate the themes/findings? Was each quotation identified? e.g. participant number	
Data and findings consistent	30	Was there consistency between the data presented and the findings?	
Clarity of major themes	31	Were major themes clearly presented in the findings?	
Clarity of minor themes	32	Is there a description of diverse cases or discussion of minor themes?	

Developed from: Tong A, Sainsbury P, Craig J. Consolidated criteria for reporting qualitative research (COREQ): a 32-item checklist for interviews and focus groups. *International Journal for Quality in Health Care*. 2007. Volume 19, Number 6: pp. 349 – 357

Once you have completed this checklist, please save a copy and upload it as part of your submission. DO NOT include this checklist as part of the main manuscript document. It must be uploaded as a separate file.

For peer review only
